# An Arabidopsis Natural Epiallele Maintained by a Feed-Forward Silencing Loop between Histone and DNA

**DOI:** 10.1371/journal.pgen.1006551

**Published:** 2017-01-06

**Authors:** Astrid Agorio, Stéphanie Durand, Elisa Fiume, Cécile Brousse, Isabelle Gy, Matthieu Simon, Sarit Anava, Oded Rechavi, Olivier Loudet, Christine Camilleri, Nicolas Bouché

**Affiliations:** 1 Institut Jean-Pierre Bourgin, INRA, AgroParisTech, CNRS, Université Paris-Saclay, Versailles, France; 2 Department of Neurobiology, Wise Faculty of Life Sciences and Sagol School of Neuroscience, Tel Aviv University, Tel Aviv, Israel; Gregor Mendel Institute of Molecular Plant Biology, AUSTRIA

## Abstract

The extent of epigenetic variation is currently well documented, but the number of natural epialleles described so far remains very limited. Determining the relevance of epigenetic changes for natural variation is an important question of research that we investigate by isolating natural epialleles segregating in Arabidopsis recombinant populations. We previously described a genetic incompatibility among Arabidopsis strains based on the silencing of a gene involved in fitness. Here, we isolated a new epiallele resulting from the silencing of a transfer-RNA editing gene in an Arabidopsis accession from the Netherlands (Nok-1). Crosses with the reference accession Col-0 show a complete incompatibility between this epiallele and another locus localized on a different chromosome. We demonstrate that conversion of an unmethylated version of this allele occurs in hybrids, associated with modifications of small RNA populations. These epialleles can also spontaneously revert within the population. Furthermore, we bring evidence that neither METHYLTRANSFERASE 1, maintaining methylation at CGs, nor components of RNA-directed DNA methylation, are key factors for the transmission of the epiallele over generations. This depends only on the self-reinforcing loop between CHROMOMETHYLASE 3 and KRYPTONITE, involving DNA methylated in the CHG context and histone H3 lysine 9 methylation. Our findings reveal a predominant role of this loop in maintaining a natural epiallele.

## Author Summary

Epialleles are gene variants based on epigenetic marks stably transmitted between generations. Most of the known epialleles existing in the wild were described in plant populations but very few are associated with phenotypes or agronomical traits. In this study, we isolated a new natural epiallele resulting from the silencing of a RNA editing gene essential for plants. We demonstrated that an incompatibility between two Arabidopsis strains depending on this epiallele, is based on DNA methylation of cytosines, an epigenetic mark influencing gene function. In F1 hybrids, obtained by crossing the incompatible parental lines, unmethylated versions of the allele can be converted to methylated ones. The epiallele can also spontaneously revert in very rare cases, within the population. The methylation status of this epiallele can therefore potentially change within the population and is maintained in a metastable state. Indeed, two enzymes promoting histone or DNA methylation, respectively, and acting in loop, are involved in maintaining the epiallele in natural populations, over generations.

## Introduction

Epialleles, identified in different organisms and predominantly in plants (reviewed by [1]), are gene variants based on epigenetic marks stably transmitted between generations. Most of the plant epialleles described so far depend on DNA methylation of cytosines, an epigenetic mark influencing the way genes are transcribed (reviewed by [2]). Few reports depict plant natural epialleles associated with phenotypes or agronomical traits. For instance, *Colourless non-ripening* is a tomato natural epivariant corresponding to a hypermethylated version of an SBP-box gene and resulting in the silencing of the gene. Ripening is inhibited in plants carrying the *Cnr* epimutation, and consequently fruits are colorless [3]. Similarly, the *peloric* phenotype in *Linaria vulgaris* is due to methylation variations [4]. In melon, spreading of DNA methylation from a transposon influences the transcription of the *CmWIP1* gene that controls sex determination [5]. Several epialleles were also identified in rice, all of them presenting severe morphological phenotypes: *Epi-d1* [6], *Epi-df* [7] and *Epi-rav6* [8]. In natural populations of Arabidopsis, epivariants of *QQS* [9] or *PAI* [10] were similarly described, although no specific phenotypes associated with the epiallelic version of the gene were discovered, beyond the change in transcription. Other epivariants arose only in mutants with drastically modified epigenomes such as *deficient in dna methylation1* (*ddm1*) encoding a chromatin-remodelling factor [11]. In our previous study [12], we demonstrated that an incompatibility between two Arabidopsis accessions is mediated through natural DNA methylation variation. In the progeny of a cross between the reference accession Col-0 and an accession from Tajikistan called Shahdara (Sha), a particular allelic combination at two loci, localised on chromosome 4 and 5, respectively, is counter-selected and very rare. In Sha, both loci carry duplicated *FOLT* genes encoding transporters of folate essential for fertility. *FOLT1*, localised on chromosome 5, is silenced by DNA methylation, while the chromosome 4 locus comprises a complex genomic rearrangement including functional and truncated copies of a paralogue, *FOLT2*. Col-0 contains only an unmethylated version of *FOLT1* at chromosome 5. Consequently, after a cross between Col-0 and Sha, plants inheriting only the methylated *FOLT1* epiallele from Sha are mostly sterile because the function of *FOLT* is missing. Since we discovered small RNAs (sRNAs) targeting the *FOLT* genes in Sha, we reasoned that *FOLT1* is silenced by RNA-directed DNA methylation (RdDM). We also found that unmethylated *FOLT1* alleles can be converted *in trans* when *FOLT2* copies are present at the other locus [12].

Different key steps, involving two plant-specific polymerases, define a feed-forward loop that controls the RdDM pathway in Arabidopsis (recently reviewed by [13]): the RNA polymerase IV (PolIV) is first recruited at methylated DNA and transcribes them to single-stranded RNA. The complementary strand of this transcript is then rapidly synthesized by the RNA-DEPENDENT RNA POLYMERASE 2 (RDR2), cleaved by DICER-LIKE 3 (DCL3) into sRNAs of 24-nt that are guiding ARGONAUTE 4/6 (AGO4/6) to regions transcribed by another RNA polymerase (PolV), finally attracting DNA methyltransferases. In addition to the RdDM, other pathways are involved in maintaining the methylation.

In plants, methylation occurs at cytosines in CG, CHG and CHH (where H = A, T, C) contexts, and the different proteins controlling these pathways are now well characterized, particularly in Arabidopsis. DNA METHYLTRANSFERASE 1 (MET1) maintains CG methylation, while CHROMOMETHYLASE 2 (CMT2) and CMT3 are DNA methyltransferases responsible for CHH and CHG maintenance, respectively. Methylation on lysine 9 of histone H3 (H3K9me) and non-CG DNA methylation are tightly correlated. CMT2 and CMT3 are recruited to regions enriched in H3K9me [14, 15] and in a reciprocal way, H3K9 histone di-methyltransferases, predominantly KRYPTONITE (also known as SUVH4 and hereafter called KYP), bind CHG-methylated cytosines through their SRA domains [16] to methylate nearby histones. Thus, CMTs and KYP participate in a self-reinforcing loop between DNA and histone methylation, essential to silence transposons and repeated sequences, but deleterious to genes [17, 18]. Recent data point toward the contribution of CMT2 in epigenetic variation of natural Arabidopsis populations [19, 20].

Here, we describe the molecular mechanism that underlies a new allelic incompatibly identified in natural populations of Arabidopsis, which is based on the silencing of a gene homologous to the yeast *transfer RNA ADENOSINE DEAMINASE 3* (*TAD3*), that is crucial for transfer RNA (tRNA) editing [21]. We identified spontaneous revertants and moreover determined that unmethylated alleles could be converted in hybrids, associated with a complete change in the population of sRNAs targeting this region. Finally, we provide evidence that only CMT3 and KYP are essential to maintain the epiallele over generations.

## Results

### The loci involved in the allelic incompatibility

A recombinant inbred lines (RIL) population was generated from a cross between two accessions of *Arabidopsis thaliana*, Nok-1 and Col-0 (http://publiclines.versailles.inra.fr/). In this population comprising 222 genotyped individual lines, linkage disequilibrium (LD) analyses revealed two physically unlinked loci segregating dependently from each other: a locus at ~14–15 Mb near the centromere of chromosome 1 appears as in significant LD with a locus at ~ 8.5 Mb on chromosome 5. Indeed, one of the four homozygous allelic combinations expected from the segregation of two independent loci—the combination of genotypes Col-0 at the chromosome 1 locus and Nok-1 at the chromosome 5 locus, named “incompatible”—is missing in the RIL population. A similar allelic incompatibility involving colocalizing loci was also observed in a different RIL set obtained by crossing Col-0 and Est-1, as documented previously [22].

**Fig 1:**
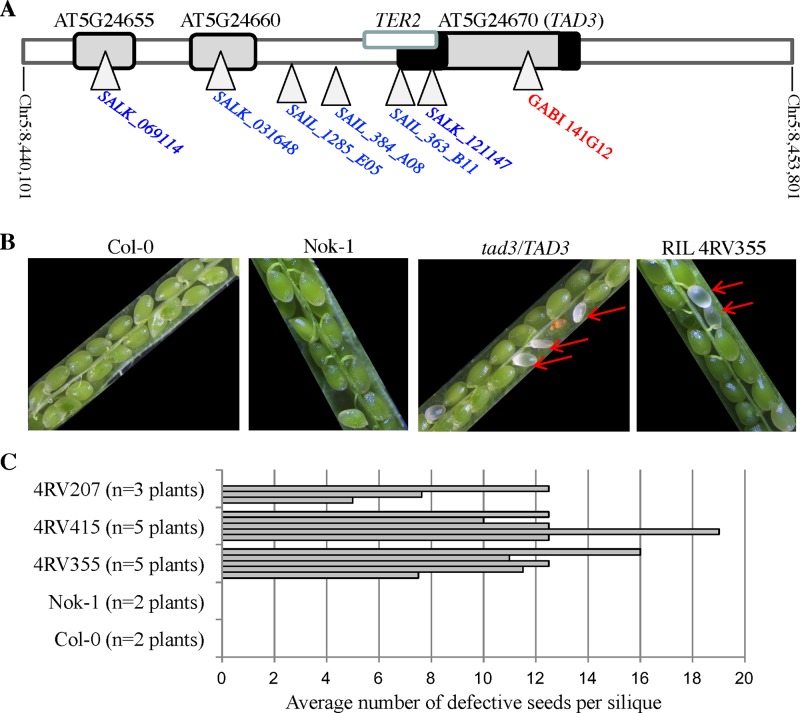
*TAD3* is the candidate gene for the incompatibility between Nok-1/Est-1 and Col-0. (A) The mapping interval localized on chromosome 5 in Col-0 contains four predicted genes. *TER2* encodes a non-coding RNA associated with the telomerase [24]. *AT5G24655* and *AT5G24660* are homologous genes involved in sulfur response [53]. *AT5G24670* encodes an enzyme involved in tRNA editing (*TAD3*) and GABI_141G12 was described [23]. The T-DNA positions in individual mutants isolated are represented by triangles. We recovered homozygous mutants for lines depicted in blue, and heterozygous mutants for lines depicted in red. T-DNAs in both SALK_121147 and SAIL_363_B11 are inserted in the 5’-UTR of *TAD3*, disrupting *TER2*. UTRs of *TAD3* are represented by black boxes. (B) Siliques of a *tad3*/*TAD3* heterozygous T-DNA plant (GABI_141G12) are similar to siliques of a F8 RIL plant (4RV355) carrying Col-0 alleles at chromosome 1 and heterozygous Col-0/Nok-1 for the chromosome 5 locus. Red arrows indicate aborted seeds. (C) Number of defective seeds per silique in three different RILs (F8 generation) carrying Col-0 alleles at chromosome 1 and heterozygous Col-0/Nok-1 for the chromosome 5 locus. One to five siliques per plant were phenotyped, representing a total of 400 embryos for 4RV207, 227 embryos for 4RV355 and 565 embryos for 4RV415.

We fine-mapped the two loci involved in the allelic incompatibility using recombinant plants in the progenies of Nok-1 x Col-0 RILs that were still heterozygous for either of the two loci and fixed for the other one (S1 Fig). The two incompatible loci were restricted, respectively, to 1.7 Mb near the centromere of chromosome 1 (S1A Fig) and to 13 kb on chromosome 5 (S1B Fig). In the reference accession Col-0, the chromosome 5 interval contains four genes (Fig 1A) and shares no sequence homologies with the second interval localized on chromosome 1. We isolated T-DNA insertion lines in a Col-0 background for each of these four genes: we recovered homozygous plants for all genes, except when the T-DNA was inserted in the coding region of *AT5G24670* in the mutant line GABI_141G12 (Fig 1A). Moreover, in this line, plants heterozygous for the T-DNA insert exhibited partial seed abortion, with embryos arresting before the globular stage (Fig 1B and [23]). The same phenotype was observed in siliques of three different RILs homozygous Col-0 at chromosome 1 and heterozygous Nok-1/Col-0 at chromosome 5 (Fig 1B and 1C). Interestingly, we recovered homozygous plants when T-DNAs were inserted in the 5’-UTR region of *AT5G24670* (Fig 1A), implying that the disruption of *TER2*, producing a non-coding RNA associated with the telomerase [24], is not embryo-lethal, contrarily to *AT5G24670*. These results make *AT5G24670*, which encodes a protein homologous to the *tRNA ADENOSINE DEAMINASE 3* (*TAD3*) essential in yeast [21], an excellent candidate gene to be responsible for the incompatibility between Nok-1/Est-1 and Col-0.

### Identification of additional copies of *TAD3* in Nok-1 and Est-1

**Fig 2:**
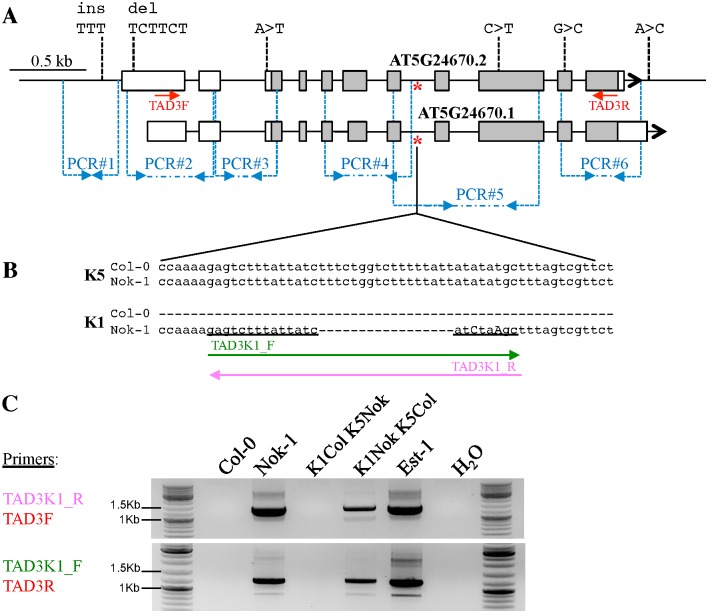
*TAD3* sequence differences between Nok-1 and Col-0. (A) *TAD3-1* polymorphisms between Col-0 and Nok-1 obtained by sequencing the whole gene (S2 Fig). The 11 exons of *TAD3-1* are represented by rectangles. The two predicted transcript isoforms of Col-0 are shown, they only differ by their UTRs (white parts of the rectangles). Compared to Col-0, Nok-1 *TAD3-1* contains an insertion (*ins*), a deletion (*del*) and four SNPs in the coding region (grey parts of the rectangles). Other SNPs, not presented here, were also found in introns (S2B Fig). The A>T change in the 5’-UTR is the only one shared by both Est-1 and Nok-1. The red star indicates the position of the 17 bp deletion found in other copies in Nok-1, used to design primers specific for Nok-1 *TAD3* on chromosome 1 (B). The sequence corresponding to these primers is underlined. (C) Nok-1 and Est-1 are carrying extra copies of *TAD3* on chromosome 1. PCR amplification on genomic DNAs extracted from plants with the indicated genotypes. *K1NokK5Col* corresponds to RILs fixed for the Nok-1 allele at chromosome 1 and for the Col-0 allele at chromosome 5. *K1ColK5Nok* corresponds to revertant plants fixed for the Col-0 allele at chromosome 1 and for the Nok-1 allele at chromosome 5 (see Fig 4). Primers are as described in (B) for the primers specific for chromosome 1 and (A) for the other primers.

To understand whether Nok-1, Col-0 and Est-1 were carrying different genetic variants of *TAD3*, we amplified the whole gene plus 1.4 kb before the ATG in Nok-1, Est-1 and Col-0 (S2A Fig). Sequencing this unique amplicon of ≈3.7 kb revealed several differences between Nok-1 or Est-1 and the Col-0 genomic sequence publicly available. In particular, Nok-1 has (1) an insertion of three Ts and a deletion of TCTTCT within the promoter sequence, (2) one SNP (A>T) in the 5’-UTR, (3) two SNPs changing two amino acids in the gene body (Fig 2A). We also found two other SNPs localized in an intron (S2B Fig). The SNP localized in the 5’-UTR was the only polymorphism common to both Est-1 and Nok-1. The TCTTCT deletion was mapped at chromosome 5 during the fine-mapping process, therefore, this copy, hereafter called *TAD3-1*, is located on chromosome 5 in Nok-1 and Est-1 as in Col-0 (S2B Fig).

When we amplified *TAD3* on genomic DNA with primers anchored within the coding sequence, we detected different copies in both Nok-1 and Est-1, in contrast to Col-0. By cloning and sequencing the corresponding PCR products, we found other copies in Nok-1 and Est-1 that present a number of polymorphisms with respect to *TAD3-1*, including a 17 bp deletion within an intron (Fig 2A, red star, and Fig 2B). Using primers surrounding this deletion, we maped these extra copies in the Nok-1 x Col-0 RILs and showed that they are located at the incompatible locus on chromosome 1 (S1 Table). We designed new primers (named *TAD3K1_F/R*; Fig 2B) overlapping this deletion to specifically amplify the *TAD3* genes localized on chromosome 1 (Fig 2C), revealing two other copies carrying SNPs specific for chromosome 1 (S1 Text). Therefore, Nok-1 and Est-1 are carrying at least two copies of *TAD3* at chromosome 1 (named *TAD3-2* and *TAD3-3)* and one at chromosome 5. All of them are polymorphic between them and compared to the unique *TAD3-1* gene of Col-0.

### *TAD3* is differentially expressed in Nok-1, Est-1 and Col-0

We first analyzed the expression of the genes using primers anchored in the coding region of all copies: two mRNAs were detected in both Nok-1 and Est-1 in contrast to Col-0 (S3A Fig). The two *TAD3* cDNAs amplified in Nok-1 and Est-1 were isolated and sequenced. Compared to Nok-1 and Est-1 *TAD3-1* sequences of chromosome 5, both are carrying seven SNPs (S3B Fig), identical to those identified on the *TAD3-2* genomic sequence mapped on chromosome 1 (S1 Text), indicating that the corresponding mRNAs are transcribed from this paralog.

**Fig 3:**
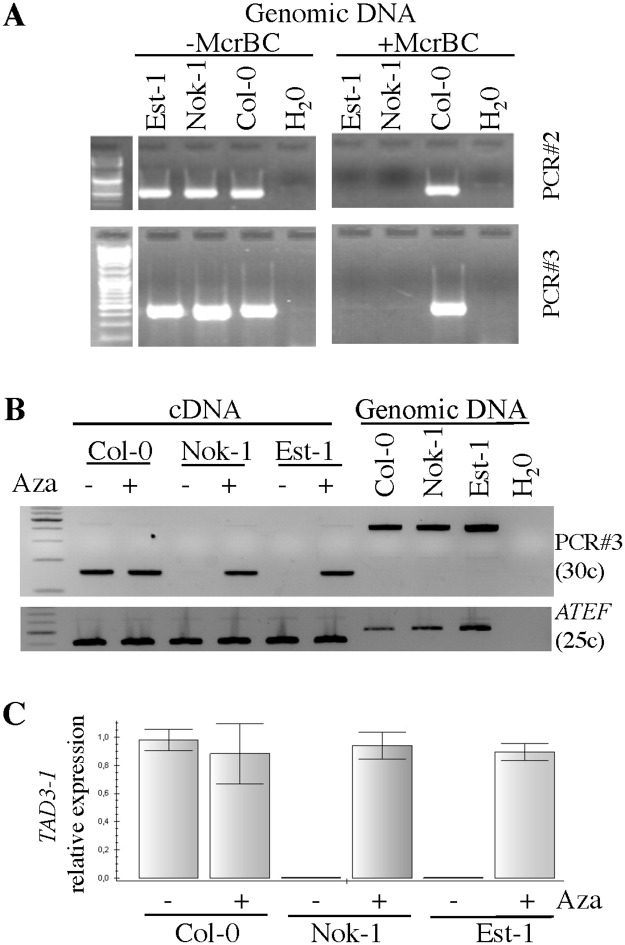
*TAD3-1* is methylated in Nok-1 and Est-1, preventing its expression. (A) DNA methylation of *TAD3-1* 5’-UTR analysed by digesting the indicated genomic DNA with *McrBC* followed by PCR amplification. Regions amplified correspond to *PCR#2* and *PCR#3* (Fig 2A) and are specific for *TAD3-1* (S4 Fig). (B) *TAD3-1* is silenced in Nok-1 and Est-1. Plants were grown on medium containing 0 (-) or 10 (+) μg/ml of 5-aza-2’deoxycytidine (*Aza*) for seven days. RNAs of plants were extracted and cDNAs were amplified using primers corresponding to PCR#3 (Fig 2A), specific for chromosome 5. *ATEF* amplifications served as controls. (C) *TAD3-1* qRT-PCR analyses using PCR#3 primers that are specific for chromosome 5 and plants described in (B).

We designed specific primers (S4 Fig) to study the expression of *TAD3-1* in Nok-1, Est-1 and Col-0. In Col-0, *TAD3-1* is transcribed in two isoforms (*AT5G24670*.*1* and .*2*), differing only by their UTRs (Fig 2A). After RT, we amplified a region common to the two transcripts in both Col-0 and Nok-1 (PCR#3), and we found that the Nok-1 *TAD3-1* transcripts were expressed at very low levels compared to Col-0 (S5 Fig). qRT-PCR analyses using the same primers confirmed that *TAD3-1* is about 30 times more expressed in Col-0 than in Nok-1 and Est-1 (see Col-0, Nok-1 and Est-1 controls in Fig 3C). Thus, in Nok-1 and Est-1, *TAD3-1* is expressed at very low levels, suggesting that their functional copy is localized on chromosome 1.

Our data point toward a duplication carrying at least two extra copies of *TAD3* on chromosome 1 in both Nok-1 and Est-1. In these two accessions, one chromosome 1 copy is transcribed whereas *TAD3-1* is not. Consequently, seeds that are Col-0 at the chromosome 1 locus and Nok-1 at the chromosome 5 locus have no functional copy of this essential gene and then abort. Altogether, these results indicate that *TAD3* is the gene involved in this allelic incompatibility between the Arabidopsis Nok-1/Est-1 and Col-0 accessions.

### *TAD3-*1 is differentially methylated in Nok-1 and Col-0

To determine why *TAD3-1* levels of expression are different from Col-0 in Nok-1 and Est-1, we monitored its levels of methylation in these accessions. The epigenome public data [25] indicate that, in contrast to Col-0, the region of *TAD3-1* is heavily methylated in both Nok-1 and Est-1. PCR amplification of the *TAD3-1* 5’-UTR after digestion with the methylation sensitive endonuclease *McrBC* confirmed that this region is methylated in both Nok-1 and Est-1 but not in Col-0 (Fig 3A, *PCR#2* and *#3*). To confirm that expression of *TAD3* and level of methylation are related, we grew the parental accessions on medium containing 5-aza-2’deoxycytidine, which inhibits cytosine methylation. The treatment activated the expression of *TAD3-1* in both Nok-1 and Est-1 (Fig 3B) to levels similar to those of Col-0 (Fig 3C). *TAD3-1* is thus methylated and silenced in both Nok-1 and Est-1, contrarily to Col-0 and this epiallele was hereafter referred as *tad3-1*.

Using the short indel of 7 bp that differentiates *TAD3* copies from chromosomes 1 and 5 (S1 Text), we designed primers specific for the 5’ region of the chromosome 1 *TAD3* copies (S6A Fig). No PCR products were obtained when the DNAs were digested with *McrBC* prior to the amplification, indicating that the chromosome 1 copies are methylated (S6B Fig).

### The methylation of Nok-1 *tad3-1* epiallele is reversible

**Fig 4:**
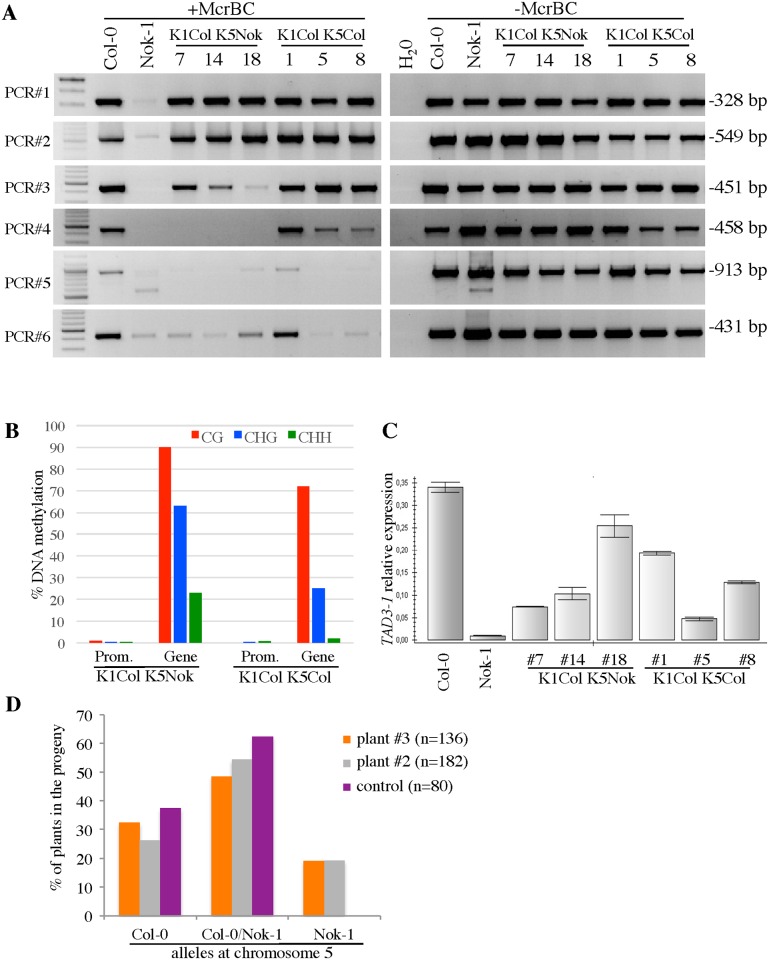
The methylation of the *tad3-1 epiallele* is reversible. (A) Genomic DNAs (300 ng) from the F9 plants indicated were digested with *McrBC (+McrBC*) and then amplified using primers specific for the regions indicated (see Fig 2A to localize the amplicons within *TAD3*). We verified the presence of the polymorphisms between Nok-1 and Col-0 (Fig 2A) by sequencing the PCR fragments obtained without *McrBC* treatment (-*McrBC*) for PCR#2, #3, #5 and #6, excluding a genetic recombination between Col-0 and Nok-1 in this region. All plants are from the progeny of an F8 revertant that was fixed Col-0 at chromosome 1 and heterozygous Col-0/Nok-1 at chromosome 5. *K1ColK5Nok* are F9 plants fixed Col-0 at chromosome 1 and Nok-1 at chromosome 5. *K1ColK5Col* are F9 plants fixed Col-0 at both chromosomes. For the Nok-1 plants, only PCR#2 and #3 are specific for chromosome 5. (B) The methylation rates within the promoter and the gene body of *TAD3* were determined in plants described in (A). Data were obtained by amplifying the regions indicated, namely ‘*Prom*.’ for the promoter and ‘*Gene’* for the gene body, after bisulfite conversion (S7 Fig). (C) Expression of *TAD3* analyzed by qRT-PCR in plants described in (A) using primers corresponding to PCR#3 (Fig 2A), specific for chromosome 5. (D) Segregation of the Nok-1 and Col-0 alleles at chromosome 5 in the progeny of the revertant. We genotyped the progeny of two F9 plants (#3 and #2), descending from the revertant, and fixed Col-0 at chromosome 1 and heterozygous Col-0/Nok-1 at chromosome 5. The control is an F9 plant fixed Col-0 at chromosome 1 and heterozygous Col-0/Nok-1 at chromosome 5, coming from a lineage independent of the revertant. The numbers of plants genotyped are indicated in parentheses.

In the course of this study, we unexpectedly identified, in the progeny (n = 60) of one F8 plant fixed Col-0 at chromosome 1 and heterozygous Nok-1/Col-0 at chromosome 5, 21% of plants carrying the incompatible allelic combination. We hypothesized that this F8 plant or one of its ancestors was already carrying a reverting *tad3-1* epiallele since revertants were not detected within the 7,872 F7 plants genotyped to map the *TAD3* loci. To determine the molecular basis of this spontaneous reversion, we examined the methylation pattern of *TAD3-1* in the F9 progeny of this plant. Different parts of the gene were amplified from three plants fixed Col-0 at chromosome 1 and Nok-1 at chromosome 5 and three sibling plants fixed Col-0 at both loci after digestion with *McrBC* (Fig 4A). In all plants carrying the incompatible combination (*i*.*e*. K1ColK5Nok), the promoter and the 5’-UTR were not methylated anymore, contrarily to Nok-1 (Fig 4A, PCR#1–3), while the second part of the gene and the 3’-UTRs remained methylated (Fig 4A, PCR#4–6). By sequencing the promoter region and the last part of the gene after bisulfite conversion, we confirmed that the promoter was not methylated and we determined that cytosines were methylated in all contexts in the gene body (Fig 4B and S7 Fig). In these plants, qRT-PCR analyses revealed that *TAD3-1* is expressed at levels intermediate between the Col-0 and Nok-1 alleles (Fig 4C). We concluded that the silencing of the methylated Nok-1 *tad3-1* epiallele is reversible, although this seems to be a rare event. In revertants, the promoter and the first part of the gene, including the 5’-UTR, are demethylated contrarily to the rest of the gene, representing very few nucleosomes. Thus, it is likely that different *TAD3-1* epiallelic versions are present in Arabidopsis and that the status of this epiallele can change within few generations. We genotyped the progeny of two F9 plants fixed Col-0 at chromosome 1 and heterozygous at chromosome 5 (Fig 4D). 19% of the F10 plants had the incompatible allelic combination, indicating that the reverting *tad3-1* epiallele is stably transmitted to the next generations.

### The Col-0 *TAD3-1* allele is convertible in hybrids

To further understand how the *TAD3-1* methylation is established, we crossed Col-0 and Nok-1 and we determined the methylation states of *TAD3-1* in hybrids by sequencing their genomic DNA after bisulfite conversion. We focused our analysis on the promoter, a region appearing to be essential for the silencing of *TAD3-1*, as shown above.

**Fig 5:**
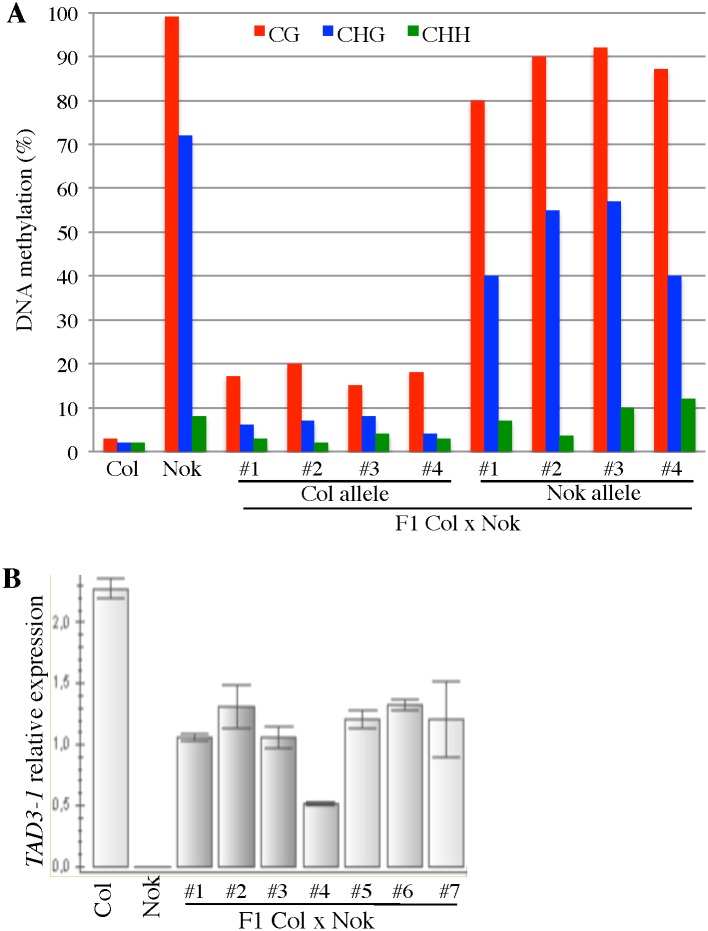
Unmethylated *TAD3-1* alleles are converted in hybrids. (A) Methylation rates within the promoter of *TAD3-1* in four hybrids obtained by crossing Col-0 and Nok-1 (Plants #1 to #4). Data (S9 Fig) were obtained by amplifying from leaf genomic DNA the promoter region corresponding to PCR#9 (S8 Fig), differentiating the Col-0 or Nok-1 alleles with the TTT insertion. (B) Expression of *TAD3-1* determined by qRT-PCR in leaves of seven different hybrids (Plants #1 to #7) including the four hybrids shown in (A).

To distinguish the Col-0 and Nok-1 *TAD3-1* alleles, we sequenced one polymorphic region (S8 Fig) in the promoter containing three additional Ts in Nok-1 compared to Col-0 (Fig 2A). In F1 hybrid plants, the Nok-1 allele of the *TAD3-1* promoter is methylated in the three cytosine contexts at levels comparable to those of Nok-1 (Fig 5A). However, we showed that the Col-0 allele gained methylation in both CG (5 to 6 times more compared to the Col-0 parent) and CHG (2 to 4 times more compared to the Col-0 parent) contexts (Fig 5A and S9 Fig). This result indicates that the Col-0 allele has been *de novo* methylated in hybrid plants. F1s obtained from both Nok-1 x Col-0 and Col-0 x Nok-1 reciprocal crosses gave similar results (S9 Fig). We then examined whether the newly acquired methylation of the Col-0 allele in F1s could interfere with the expression of *TAD3-1*. Pyrosequencing analyses using the SNP A>T in the 5’-UTR region (Fig 2A) confirmed that the *TAD3-1* Nok-1 allele is not expressed in F1s (Table 1 and S10 Fig). Additionally, expression analyses by qRT-PCR revealed that the Col-0 allele is expressed in leaves of F1s at levels intermediate between the parents (Fig 5B), which is the expected expression level when only one allele is transcribed. These results imply that the methylation gained in one generation by the Col-0 allele has a minor effect on the expression of *TAD3*.

**Table 1:** Expression profiles of *TAD3-1* alleles in Col-0 x Nok-1 hybrids determined by pyrosequencing.

		A [Col-0]	T [Nok-1]
Genomic DNA	Col-0	97	3
	Nok-1	5	95
	F1	53	47
Leaf cDNAs	Col-0	98	2
	Nok-1	no amplification	
	F1.1	97	3
	F1.2	97	3
	F1.3	98	2
	F1.4	98	2
	F1.5	97	3
	F1.6	96	4
	F1.7	99	1
Flower cDNAs	Col-0	99	1
	Nok-1	8	92
	F1.1	98	2
	F1.2	99	1
	F1.3	100	0
	F1.4	98	2
	F1.5	97	3
	F1.6	98	2
	F1.7	93	7

The fragment amplified corresponds to PCR#3 (Fig 2A) and is specific for *TAD3-1* (S4 Fig). Results are presented as percentages of the nucleotide found for the SNP (“*A*” corresponding to the Col-0 allele or “*T*” corresponding to the Nok-1 allele) in the indicated samples. F1 plants are described in Fig 5. Results are a mean of ratio found for two technical repeats per sample except for F1 DNAs for which four technical replicates were done. The pyrograms obtained are presented in S10 Fig.

### A small RNA population targets massively the region upstream of *TAD3-1* in hybrids

**Fig 6:**
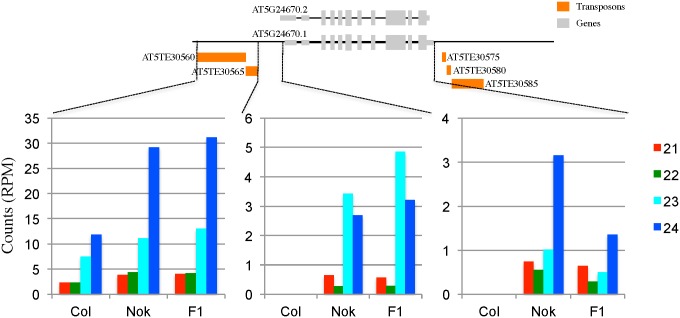
Sequence length distribution of the small RNAs over the *TAD3-1* locus. sRNA reads were counted in three different genomic regions: the first one (Chr5: 8,446,240–8,447,463) comprises two transposons upstream of *TAD3-1*, the second includes the promoter of *TAD3-1* (Chr5: 8,447,463–8,447,954) and the last one corresponds to the *TAD3-1* gene (Chr5: 8,447,954–8,451,218). Transposable elements are depicted in orange and *TAD3-1* in grey. Counts are given in reads per million of mapped reads. The precise distribution of sRNA reads is described in S11 Fig.

To determine whether an RdDM process mediated by sRNAs targets *TAD3*, we profiled, by deep sequencing, the sRNA populations of Col-0, Nok-1 and their F1 hybrid, mapping them to the Col-0 reference genome (Fig 6 and S11 Fig). In Col-0, we only detected 23/24-nt sRNAs corresponding to transposons localized upstream of the *TAD3-1* gene. In both Nok-1 and the F1, the number of 24-nt sRNAs matching these transposons increased by 2.5 times compared to Col-0. In addition, sRNAs, mostly 23/24-nt, mapping to the region localized between the transposons and the 5’-UTR of *TAD3-1* were detected in Nok-1 and the F1. In comparison, we found a limited number of sRNAs dispatched along the *TAD3-1* sequence in both the F1 and Nok-1. Thus, in both the F1 and Nok-1, 23/24-nt sRNAs matching the regions upstream of *TAD3-1* are more abundant than in Col-0. We hypothesize that the sRNAs, which cover this entire region in Nok-1 and the F1, potentially initiate the methylation through the RdDM pathway. It would be intriguing to examine in future studies whether the precursors of these sRNAs originate in *trans* or in *cis*.

### Maintenance of the *tad3-1* epiallele depends on both CMT3 and KYP

To decipher the molecular mechanisms involved in maintaining the *tad3-1* epiallele, we determined whether the incompatibility depends on particular epigenetic pathways. To this end, we crossed Nok-1 with several mutants (in a Col-0 background), which are impacted in the maintenance or the establishment of different epigenetic marks. The resulting hybrids were then back-crossed with the same mutants to obtain plants that were fixed for the mutation and heterozygous Nok-1/Col-0 at both *TAD3* loci (S12 Fig). We then followed the segregation of both loci in the selfed progenies of these plants by genotyping. As a control, we genotyped 337 F2 plants from the Nok-1 x Col-0 F1 progeny and we confirmed that Col-0 and Nok-1 alleles segregated as expected, that is missing the incompatible allelic combination (Col-0 at chromosome 1 and Nok-1 at chromosome 5; Table 2). We obtained similar results for all mutants involved in RdDM pathways, namely mutants of the DNA-dependent RNA polymerases PolIV and PolV, AGO4 and RDR2 (Table 2). Comparable results were obtained in a *met1-1* background (Table 2), implying that the *tad3-1* epiallele is maintained independently of the RdDM or MET1 pathways. Nevertheless, we found plants with an 'incompatible' allelic combination in both *cmt3* and *kyp* backgrounds (Table 2).

**Table 2:** Segregation analysis of the two *TAD3* loci in different epigenetic mutant backgrounds.

K1K5 genotype		% of each genotypic class								
	Expected	No mutation	*pol IV*	*pol V*	*rdr2*	*ago4-2*	*Plant #1* *met1-1*	*Plant #2* *met1-1*	*cmt3-11*	*kyp*
CC	6	7	9	8	4	7	5	7	10	2
CH	13	13	13	18	14	6	2	3	13	8
**CN**	**6**	**0**	**0**	**0**	**0**	**0**	**0**	**0**	**3**	**3**
NN	6	9	6	8	4	13	10	9	8	7
NH	13	12	17	18	14	17	28	24	7	15
NC	6	10	13	3	4	13	16	8	5	8
HN	13	12	14	8	10	7	7	10	9	16
HH	25	24	20	30	31	21	14	22	35	30
HC	13	15	9	7	17	16	18	17	9	10
Number of plants	100	337	127	119	134	150	138	374	134	165

Segregation analysis in the progenies of mutants heterozygous for the *TAD3* alleles at both chromosomes 1 and 5, as described in S12 Fig. In the chromosome 1 chromosome 5 (*K1K5 genotype*) genotype column, “*C*” indicates that the Col-0 allele is fixed, “*N*” indicates that the Nok-1 allele is fixed and “*H*” indicates that both alleles are present at the heterozygous state. All plants were genotyped for their respective mutations, namely *nrpd1a-4* (*pol IV*), *nrpe1-11* (*pol V*), *rdr2-2*, *ago4-2*, *met1-1*, *cmt3-11* and *kyp*. The incompatible combination (*CN*), represented in bold, only appears in *cmt3* or *kyp* backgrounds. The percentage of each genotypic class for two genetically unlinked loci segregating independently is presented in the column “*Expected*”. The percentage of each genotypic class obtained for the two *TAD3* loci after a cross between Col-0 and Nok-1 is presented in the column “*No mutation*”. The total number of plants genotyped is indicated. The progenies of two different plants in the *met1-1* background were genotyped.

**Fig 7:**
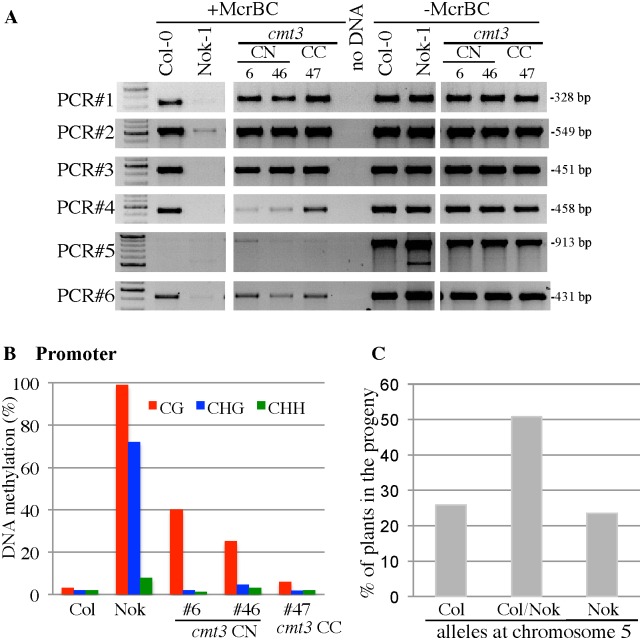
Methylation patterns of *TAD3-1* in incompatible plants carrying a *cmt3* mutation. (A) Methylation patterns of *TAD3-1* in incompatible plants carrying a *cmt3* mutation. Genomic DNAs (300 ng) were digested with *McrBC (+McrBC*) and then amplified using primers specific for the PCR fragments indicated (see Fig 2A to localise the amplicons within the *TAD3-1* gene). Plants noted “*CN*” or “*CC*” are all in the *cmt3-11* background. “*C*” indicates that the Col-0 allele is fixed, “*N*” indicates that the Nok-1 allele is fixed. For the Nok-1 plants, only PCR#2 and #3 are specific for chromosome 5. (B) Methylation rates within the promoter regions in incompatible plants carrying a *cmt3-11* mutation. Data (S13 Fig) were obtained by amplifying from leaf genomic DNA the promoter region corresponding to PCR#7 (S7A Fig). The numbers indicate the plants analyzed, as shown in (A). In a *cmt3* background, the allele inherited from Nok-1 at chromosome 5 becomes specifically hypomethylated in the CHG context compared to the Nok-1 parent (plants #6 and #46). (C) Segregation of the Nok-1 and Col-0 alleles at chromosome 5 in the progeny (n = 128) of a *cmt3* mutant. The progeny of a plant fixed Col-0 at chromosome 1 and heterozygous Col-0/Nok-1 at chromosome 5 and sibling of the plants presented on (A) and (B) were genotyped.

To further determine the methylation patterns of *TAD3-1* in this context, we extracted genomic DNAs from 'incompatible' plants obtained in the *cmt3* background and we amplified different parts of the gene, after digestion with *McrBC* (Fig 7A). The profiles of methylation obtained in a *cmt3* mutant were similar to the ones obtained in Col-0, confirming that CMT3 is necessary to maintain Nok-1 *tad3-1* epiallele methylation. Sequencing the promoter regions after bisulfite conversion revealed that the non-CG methylation from the Nok-1 allele is missing in these *cmt3* backgrounds (Fig 7B), while the level of CG methylation was intermediate between Col-0 and Nok-1 (Fig 7B and S9 Fig). In the progeny (n = 128) of one *cmt3* plant fixed Col-0 at chromosome 1 and heterozygous Nok-1/Col-0 at chromosome 5, 23% of the plants were carrying the incompatible allelic combination (Fig 7C), showing that CMT3 maintains *tad3-1* over generations.

## Discussion

Very few metastable epialleles were identified in natural populations, mostly in plants, and even less are associated with phenotypic effects. In the present study, we identified a new epivariant of the *TAD3* gene in Arabidopsis. *TAD3* encodes a tRNA-editing deaminase, converting adenosine to inosine, and is essential for plants [23, 26]. Col-0 contains a unique functional copy of the gene localized on chromosome 5. Other accessions, like Nok-1 or Est-1, have paralogs on chromosome 1, including transcriptionally active copies, while their chromosome 5 copy, methylated and not expressed, corresponds to a new epiallele not described before.

Similarly to other epivariants like the *Peloric* mutant [4] or *Cnr* in tomato [27], we observed cases of reversion for *tad3* (Fig 4). This seems to be a rare event since we only observed it one time in the course of this study while we genotyped more than 7,800 plants to map the *TAD3* loci. Comparatively, *Cnr* fruits with reverting sectors were observed in four cases out of thousands of fruits [27]. Thus, the reversion of both *Cnr* and *tad3* epialleles are singular events. Since we found revertants only in the progeny of one F8 plant from a RIL population, this could indicate that fully reverting the methylation of *TAD3* takes several generations to occur. The (meta-)stability of these epialleles are very interesting to study in an evolutionary context, where their stochasticity and quantitative nature could modify the way these changes are inherited throughout generations [28].

### Role of the RdDM pathway and small RNAs

Transposable Elements (TEs) or repetitive sequences, like those localized upstream and downstream of the *TAD3* gene in Col-0 (Fig 6), are prone to silencing and can influence the transcription of genes. First, the spreading of epigenetic marks from the TE toward neighbouring genes localized *in cis* can be deleterious for their transcription. Not surprisingly, there are many examples of plant epialleles whose silencing depends on an adjacent TE or repeat: *Epi-1d* [6] and *Epi-rav6* [8] in rice, *CmWIP1* in melon [5], *FLOWERINGWAGENINGEN (FWA)* [29, 30] and *BONSAI (BNS)* [31] in Arabidopsis. Second, sRNAs produced from a TE or repetitive sequences can also trigger the silencing of homologous regions *in trans*, including genes, through the RdDM pathway. In a previous study [12], we identified two incompatible loci in a RIL population obtained by crossing Col-0 and Shahdara. We demonstrated that this incompatibility is based on a duplication and rearrangement of the *FOLT* gene. The Shahdara copy on chromosome 4 produces sRNAs (detected by northern blot) that cause a *FOLT* copy on chromosome 5 to be methylated and silenced *in trans*. In this case, sRNAs are abundant and homologous to the *FOLT* gene promoter and the first part of the coding region [12]. Therefore, it is likely that *FOLT* sRNAs are directly targeting the *FOLT* gene, triggering the methylation by RdDM. In the case of the *TAD3* gene, we were unsuccessful in detecting, by northern blot analyses, sRNAs homologous to the coding region in Nok-1, F1 and RILs. This was confirmed by the low level of sRNAs targeting *TAD3* identified by sequencing the whole population of sRNAs in both Nok-1 and the F1 (Fig 6 and S11 Fig). Furthermore, the 24-nt sRNAs homologous to *TAD3* are dispatched along the 3 kb of the gene and neither the 5’-UTR nor the promoter region immediately adjacent to the gene are massively targeted (S11 Fig). Therefore, the *TAD3-1* coding region is probably not directly targeted by sRNAs in the F1. On the opposite, a large amount of sRNAs found in Nok-1 and the F1 are matching the region corresponding to transposons localized upstream of *TAD3-1*, going over the edges of the transposons (S11 Fig). Therefore, we cannot exclude their importance in establishing a new epigenetic state near *TAD3-1* in F1s, leading to the progressive methylation of the Col-0 allele (Fig 5A). Indeed, several reports point toward drastic modifications of sRNA populations between hybrids and their parents, associated with changes in DNA methylation patterns [32–35]. Interestingly, regions localized in the vicinity of TEs seem to be particularity prone to changes in sRNA contents [36] and DNA methylation [35] in hybrids. Nevertheless, while sRNAs could initiate the spreading of methylation toward *TAD3-1* in an initial step, none of the genes involved in 24-nt sRNA synthesis tested in our study (Table 2) are important to maintain the *tad3-1* epiallele. We conclude that sRNAs are not essential to maintain the silencing of *TAD3-1* over generations but are potentially required in an early initiation step. Indeed, RdDM plays a critical role in controlling most of the DNA methylation interactions occurring in an Arabidopsis Col-0 x C24 hybrid [35].

### Maintenance of the *tad3* natural epiallele relies on the CMT3/KYP loop

In Arabidopsis, *FWA* and *BNS* are two examples of epialleles revealed and stabilized in hypomethylated *ddm1* mutants [31, 37]. *FWA* is an imprinted gene involved in flowering that is silenced in vegetative tissues, due to the methylation of a SINE-related sequence [29]. The lack of methylation results in expression of *FWA* and late flowering [30] and its maintenance depends on MET1 [38]. MET1 is, however, not involved in the maintenance of *TAD3-1* methylation: first, the incompatible allelic combination is absent from the progeny of two individual *met1* plants segregating the *TAD3* loci (Table 2) and second, plants are fertile when the promoter of *TAD3-1* is partially methylated in the CG-context (Fig 7B), maintained by MET1. Thus *FWA* and *TAD3* are maintained silenced via different pathways. Another possibility is that more generations are needed for *tad3-1* to revert in the *met1-1* background or that this requires a stronger *met1* allele.

In addition to *fwa* epialleles, the *bns* epiallele arose in hypomethylated mutant backgrounds due to changes of epigenetic patterns at nearby transposons. However, after several generations this resulted in an increasing DNA methylation at *BNS*, leading to its silencing [31]. Very similarly to the *tad3-1* epiallele, *bns* maintenance depends on both CMT3 and KYP, but not on the RdDM machinery [39]. Indeed, we demonstrate that CMT3 is involved in maintaining the *tad3-1* epiallele found in Nok-1 since we recovered incompatible plants in the *cmt3* mutant background (Table 2). We also obtained the same results in a *kyp* background (Table 2), providing further evidence that both CMT3 and KYP, involved in the same self-reinforcing loop [15, 16], are key factors to maintain the *tad3-1* epivariant between generations. Additionally, *BNS* is also hypermethylated when the function of INCREASE IN BONSAI METHYLATION 1 (IBM1), a histone demethylase removing H3K9 methylation, is compromised [17] and *TAD3* is likewise targeted by IBM1 [40].

Recently, the molecular bases involved in maintaining *Cnr* through generations were identified in tomato, with a major role played by CMT3 [27]. In this natural epivariant of the *LeSPL-CNR* gene, the promoter is methylated and the gene is transcriptionally silenced, leading to the non-ripening phenotype observed. Silencing *CMT3* in *Cnr*, but not *MET1*, resulted in the almost complete rescue of fruit ripening, associated with a reduction in CHG methylation at eight positions in the promoter of *LeSPL-CNR*. Therefore, together with the *tad3-1 epiallele*, both *Cnr* and *bns* are epialleles maintained by the CMT3/KYP loop. An intriguing question that remains to be answered is to determine the molecular events that break this loop, allowing the promoter of *TAD3-1* to be demethylated and transcribed again in revertants. Further studies are needed to clarify the role and the extent of this feed-forward loop between histone and DNA in maintaining natural epigenetic variation in plants.

## Materials and Methods

### Plant materials

*A*. *thaliana* RILs, HIFs and accessions were obtained from the Versailles Arabidopsis stock center (http://publiclines.versailles.inra.fr/). The following mutants were used: the GABI_141G12 T-DNA line [41], *met1-1* [42], *cmt3-11* (SALK_148381, [43]), *ago4-2* [44], *suvh4*/*kyp* (SALK_069326, [45]), *rdr2-2* (SALK_059661, [46]), *nrpd1a-4* (*pol IV*; SALK_083051, [47]) and *nrpe1-11* (*pol V*; SALK_029919, [48]).

### Genotyping

Col-0 sequences are corresponding to the TAIR10 version of the reference genome. To genotype plants, we used two molecular markers, one located within the 5'-UTR of *TAD3-1* (MSAT5.08448), amplifying 142 bp in Col-0 and 138 bp in Nok-1, and another one that co-segregates with the interval of 14–15 Mb at chromosome 1 (MSAT1.15597; S1 Fig), and amplifying 128 bp in Col-0 and less in Nok1 (≈120 bp). The GeneRuler DNA Ladder Mix (Ref SM0331, *Thermo*) is the DNA ladder used for all figures.

### *McrBC* treatment

Genomic DNA (300 ng) was digested for 8 h at 37°C with the *McrBC* enzyme (*New England Biolabs*), the same amount of undigested genomic DNA was used as control. The methylation of a region was assessed by PCR amplification (35 cycles) using 20 ng of digested or undigested genomic DNA. The primers are listed in S3 Table.

### Sequencing after bisulfite conversion

For each sample, 1 to 2 μg of genomic DNAs were extracted from leaves, using the NucleoSpin Plant II kit (*Macherey-Nagel*). DNAs were treated with bisulfite using the EpiTect Bisulfite Kit (*Qiagen*). Treated DNAs were amplified using primers listed in S3 Table. PCR fragments were then cloned in pTOPO (*Life Technologies*) and sequenced individually. Results were analyzed with the Kismeth tool [49].

### Gene expression analyses

Total RNAs were isolated from the aerial parts of 21 day-old seedlings grown *in vitro* using the RNeasy Plant Mini kit (*Qiagen*) followed by a DNAse treatment (*Fermentas*). RT-PCR was performed on 500 ng of total RNAs with the M-MLV reverse transcriptase (*Fermentas*) and cDNAs were diluted 10 times. 5 μl were used for qRT-PCRs using a CFX96 real-time PCR machine (*BioRad*) with a SYBR solution (*Eurogentec*) using primers listed in S3 Table. Expression levels were normalized against the Arabidopsis *UBC21* gene (*AT5G25760*).

### Pyrosequencing

Analyses were done as described before [50]. Briefly, pyrosequencing (PyroMark Q24; *Qiagen*) was used to estimate relative allele-specific expression in the F1. RNAs from leaves or flowers of Col-0, Nok-1 and seven different F1 plants were extracted to prepare cDNAs as described above. The region amplified corresponds to PCR#3 (Fig 2A) and is specific for *TAD3-1* (S4 Fig). Pyrosequencing reactions were performed using the SNP (A>T) localised in the 5’-UTR of *TAD3-1*. F1 genomic DNA was used for technical control to normalize the ratios against possible pyrosequencing or PCR biases. The sequencing primer is listed in S3 Table.

### Analyses of small RNA populations

sRNAs were extracted and sequenced as previously described [51]. Reads were first trimmed to discard reads shorter than 15 nt and to remove the adapter (AGATCGGAAGAGCACACGTCT). Clean reads were then aligned to the Col-0 genome (TAIR10.30) with *bowtie* allowing one mismatch and a maximum of 50 multi-mappings per read. Data were plotted with the *viRome* R package [52]. The reads were normalized to reads per million (RPM) of mapped reads. Statistics of the bioinformatics analyses are presented in S2 Table.

## Supporting Information

S1 Fig Fine mapping of the two loci involved in the incompatibility. Mapping intervals obtained on chromosome 1 (A) and 5 (B) are indicated. F7 RIL 4RV043 (heterozygous for both incompatible loci localized on chromosome 1 and chromosome 5) was first fixed Nok-1 at chromosome 5 and kept heterozygous at chromosome 1. F7 RIL 4RV355 was already heterozygous at chromosome 5 and fixed Col-0 at chromosome 1.
(PDF)

S2 Fig Sequencing of *TAD3-1*. (A) PCR amplification on genomic DNA using the primer set indicated. (B) Genotype differences between Nok-1 and Col-0 obtained by sequencing the PCR fragments shown in (A). Differences (SNPs or INDELs) are indicated. The two changes of amino acid are indicated. Polymorphic regions were sequenced at least three times. The positions of the two primers mentioned in (A) are indicated. The PCR amplicon corresponding to Est-1 was sequenced only in regions diverging between Nok-1 and Col-0. The microsatellite polymorphism (*MSAT5*.*08448*) used as a genetic marker to genotype the RILs and map the interval (S1 Fig) was identified in both Nok-1 and Col-0, indicating that the amplicons shown in (A) are specific to chromosome 5. Only one transcript is presented for *TAD3* (i.e. *AT5G24670*.*2*).
(PDF)

S3 Fig *TAD3* cDNAs detected in Nok-1. (A) Expression analysis of *TAD3* in Col-0, Nok-1 and Est-1. cDNAs were amplified using primers (described in S3 Table) anchored within the coding region. The numbers of PCR cycles are indicated. *ATEF* cDNA amplifications served as controls. (B) Region amplified corresponding to the PCRs shown in (A). (C) Sequences of the cDNA fragments shown in (A). The upper sequence corresponds to the Col-0 *TAD3-1* mRNA. The two other sequences correspond to the *TAD3-2* mRNA transcribed from chromosome 1 in Nok-1. Polymorphisms between the sequences are indicated in red: we identified seven SNPs and an insertion of 101 bp corresponding to the retention of one intron. The corresponding amino acids are shown.
(PDF)

S4 Fig PCR#2 and PCR#3 are specific to chromosome 5. PCR#2 and PCR#3 amplicons are positioned in Fig 2A. The reverse primer for PCR#2 and the forward primer for PCR#3 are overlapping a short deletion of 7 bp between chromosomes 1 and 5 (S1 Text). (A) PCR amplification on genomic DNAs extracted from plants with the indicated genotypes. *K1NokK5Col* corresponds to plants from the RIL population that are fixed for the Nok-1 allele at chromosome 1 and for the Col-0 allele at chromosome 5. *K1ColK5Nok* corresponds to revertant plants from the RIL population that are fixed for the Col-0 allele at chromosome 1 and for the Nok-1 allele at chromosome 5 (see Fig 4). (B) and (D) Sequences of the PCR fragments shown in (A). The corresponding electrophoregrams (C) and (E) are shown.
(PDF)

S5 Fig *TAD3-1* is expressed differently in Nok-1 and Col-0. Expression analysis of the *TAD3-1* copy in both Nok-1 and Col-0. The region amplified corresponds to PCR#3 (Fig 2A) and is specific for *TAD3-1* (S4 Fig). The numbers of cycles are indicated, and Col-0 genomic DNA was used as control. *ATEF* amplifications served as controls.
(PDF)

S6 Fig *TAD3* is methylated at chromosome 1 in Nok-1. (A) PCR fragments specific for chromosome 1 obtained by amplifying genomic DNAs with primers TAD3_K1Nok Forward and Reverse (S3 Table). The forward primer chromosome 1 specific is designed on a short deletion of 7 bp between chromosomes 1 and 5 (S1 Text). (B) DNA methylation of *TAD3* at chromosome 1 analysed by digesting the indicated genomic DNA (300 ng) with *McrBC* followed by PCR amplification. The region amplified corresponds to the PCR described in (A) and is specific for chromosome 1.
(PDF)

S7 Fig Methylation patterns of *TAD3* in revertant plants. (A) Schematic representation of the *TAD3* gene drawn to scale, PCR#7 and PCR#8 amplicons are positioned. (B) and (C) After bisulfite conversion of DNAs, the two regions indicated in (A) were amplified using the primers described in S3 Table. Sequences were aligned using the Kismeth tool [49]. The numbers indicate the plants analyzed, as shown in Fig 4. Cytosines are represented by circles (red: CG, blue: CHG, green: CHH; solid circles: methylated cytosines).
(PDF)

S8 Fig PCR#9 is specific for chromosome 5. (A) Schematic representation of the *TAD3* gene drawn to scale, the PCR#9 amplicon is positioned. (B) PCR amplification on genomic DNAs extracted from plants with the indicated genotypes. *K1NokK5Col* corresponds to plants from the RIL population that are fixed for the Nok-1 allele at chromosome 1 and for the Col-0 allele at chromosome 5. *K1ColK5Nok* corresponds to revertant plants from the RIL population that are fixed for the Col-0 allele at chromosome 1 and for the Nok-1 allele at chromosome 5 (see Fig 4). (C) Sequence of the PCR fragments shown in (B). The corresponding electrophoregrams (D) are shown.
(PDF)

S9 Fig Methylation patterns of *TAD3-1* in Col-0 x Nok-1 hybrid plants. After bisulfite conversion of DNAs, the regions corresponding to PCR#9 (S8 Fig) were amplified using the primers described in S3 Table. Sequences were aligned using the Kismeth tool [49]. (A) Results summarized in Fig 5A with Col-0 and Nok-1 parents and the four individual hybrids resulting from a Col-0 x Nok-1 cross. (B) Methylation patterns obtained in a reciprocal cross. Cytosines are represented by circles (red: CG, blue: CHG, green: CHH; solid circles: methylated cytosines). The amounts of methylated cytosines are indicated in percentages.
(PDF)

S10 Fig *TAD3-1* expression analysis using pyrosequencing. (A) Dispensation order used to analyze the A/T SNP. (B) Pyrograms obtained for the control DNAs. The luminescence is expressed in arbitrary unit (*a*.*u*.). Along the abscissa the dispensation order is given with controls (*E*: enzyme; *S*: substrate). The SNP position is tinted in grey with the calculated percentage of each nucleotide above. (C) Pyrograms obtained using F1 cDNAs from leaves and flowers.
(PDF)

S11 Fig Distribution of small RNA reads over the *TAD3-1* locus. sRNA content of the region corresponding to the mapping interval (Chr5: 8,440,101–8,453,801). sRNAs from Col-0, Nok-1 and the Col-0xNok-1 F1 were mapped to the Col-0 genome (TAIR10.30 version). Both sense and antisense reads were collapsed and only reads corresponding to 21 to 24-nt are plotted. Transposons are in orange.
(PDF)

S12 Fig Scheme showing crosses done to obtain the *TAD3* loci in different mutant backgrounds. Example of cross between Nok-1 and a mutant (here *nrpe1-11*) in a Col-0 background performed to obtain a plant fixed for the mutation and heterozygous at both *TAD3* loci.
(PDF)

S13 Fig Methylation patterns of the promoter regions in incompatible plants carrying a *cmt3-11* mutation. After bisulfite conversion of DNAs, the region corresponding to PCR#7 (S7A Fig) was amplified using the primers described in S3 Table. Sequences were aligned using the Kismeth tool [49]. The numbers indicate the plants analyzed, as shown in Fig 7A. Cytosines are represented by circles (red: CG, blue: CHG, green: CHH; solid circles: methylated cytosines). Results are summarized in Fig 7B. Col-0 and Nok-1 parents are shown in S9A Fig.
(PDF)

S1 Table The deletion of 17 bp maps to the incompatible locus on chromosome 1 in Nok-1. Using primers (*IND5*.*08449F* and *IND5*.*08449R*) flanking the deletion of 17 bp (Fig 2), 162 plants from the Nok-1 x Col-0 RIL population were genotyped. The shorter fragment (265 bp) is amplified only when RILs are fixed Nok-1 at chromosome 1, confirming that the deletion is associated with a *TAD3* paralog in this region.
(PDF)

S2 Table Statistics of the sRNA bioinformatics analysis. (PDF)

S3 Table List of primers. (PDF)

S1 Text Sequences of the different *TAD3* copies identified in Nok-1. SNPs/indels found between chromosomes 1 and 5 are in red. SNPs/indels found between the copies mapped at chromosome 1 are in blue. Stars indicate nucleotides that are identical between all sequences. Bold stars and sequences underlined indicate transcribed regions. Start and stop codons are in bold. The SNPs identified in the cDNAs (S3 Fig) correspond to *TAD3-2*. The deletion of 7 bp (ATCGGCT) between the copies of chromosomes 1 and 5 was used to design primers specific for chromosome 1 (Fig 3A). The reverse primer for PCR#2 and the forward primer for PCR#3 are overlapping this deletion, and are specific for chromosome 5.
(PDF)
