# Supplementary figures and images for: An Arabidopsis Natural Epiallele Maintained by a Feed-Forward Silencing Loop between Histone and DNA
Source: PLoS Genet. 2017 Jan 6;13(1):e1006551. doi: 10.1371/journal.pgen.1006551 (PMC5257005; doi:10.1371/journal.pgen.1006551)

Sup Figure S1

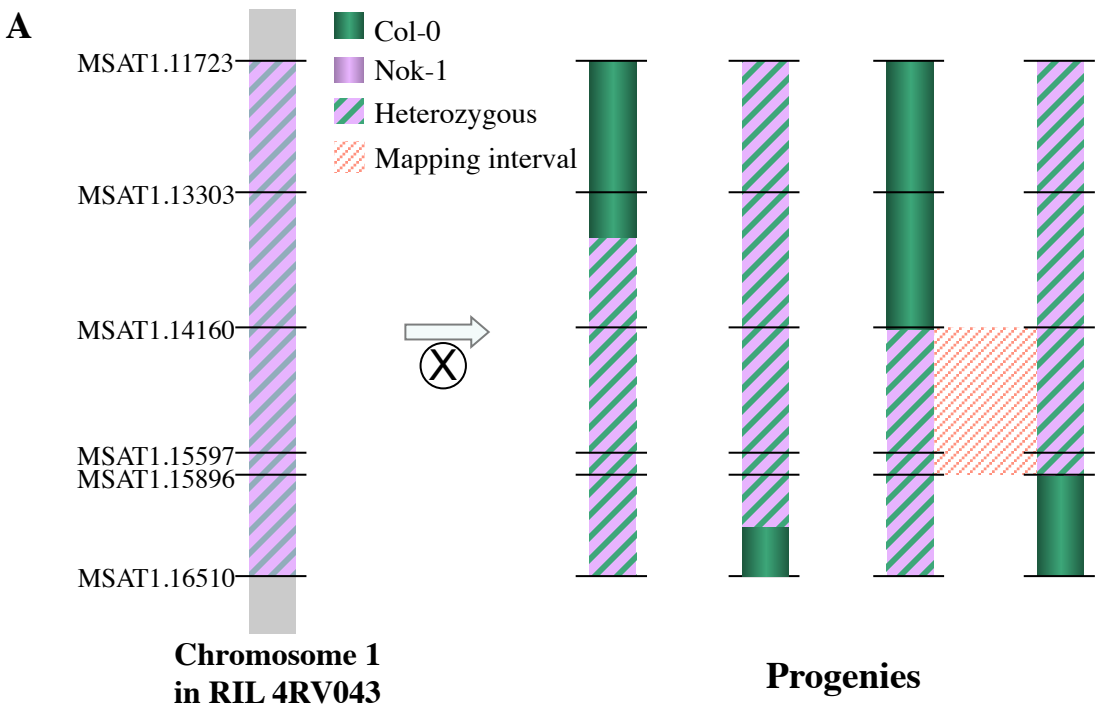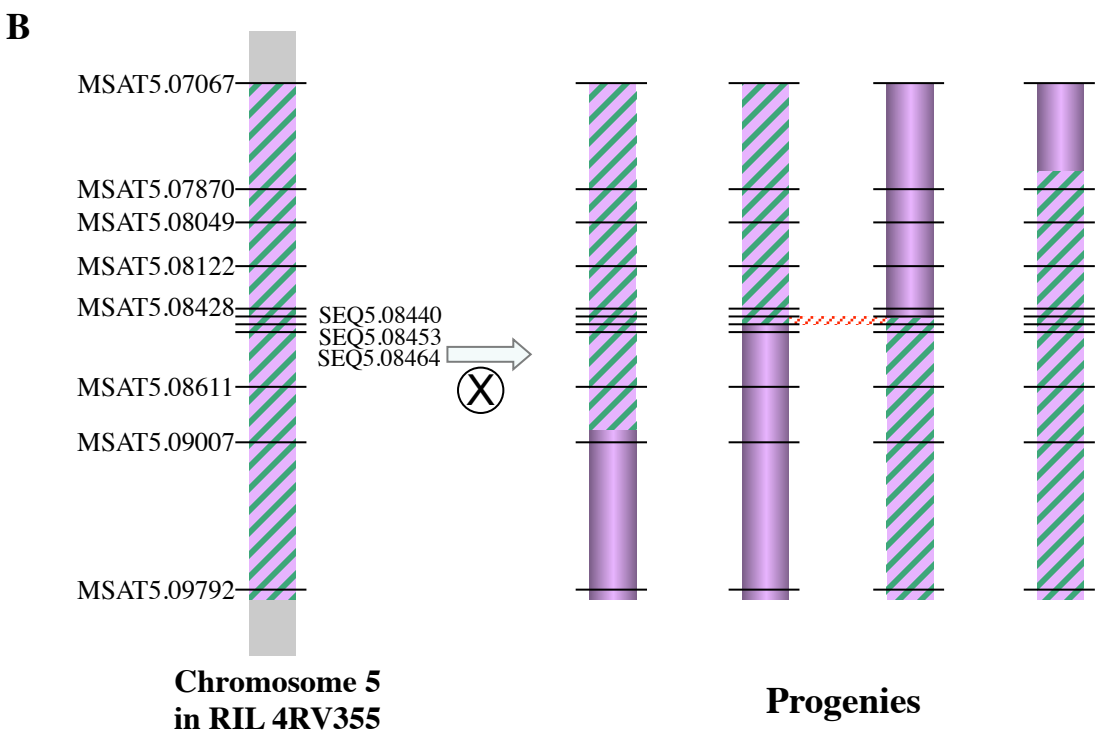

Supplement: S1 Fig — Mapping intervals obtained on chromosome 1 (A) and 5 (B) are indicated. F7 RIL 4RV043 (heterozygous for both incompatible loci localized on chromosome 1 and chromosome 5) was first fixed Nok-1 at chromosome 5 and kept heterozygous at chromosome 1. F7 RIL 4RV355 was already heterozygous at chromosome 5 and fixed Col-0 at chromosome 1. (PDF) [file pgen.1006551.s001.pdf]

Sup Figure S2

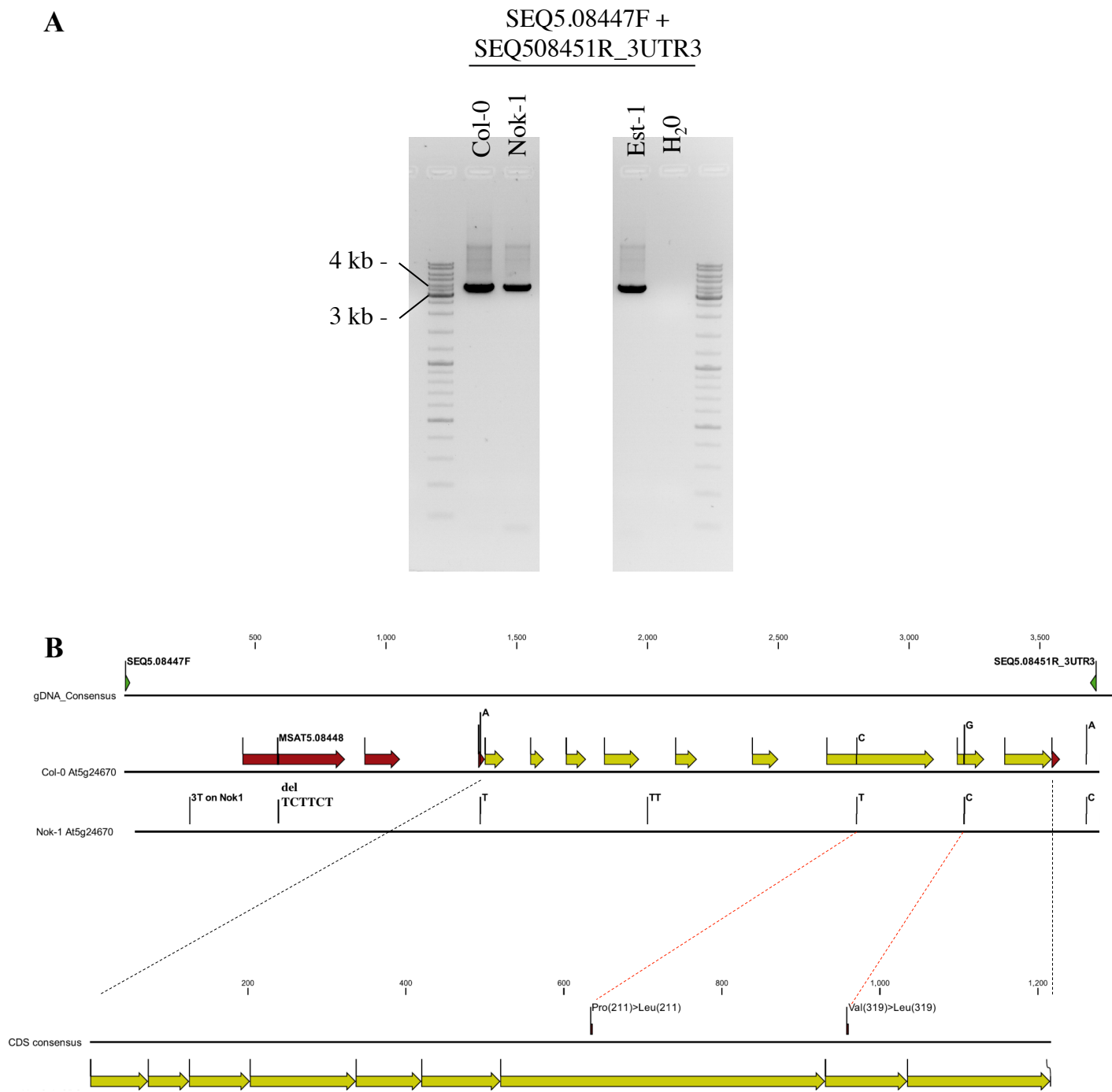

Supplement: S2 Fig — (A) PCR amplification on genomic DNA using the primer set indicated. (B) Genotype differences between Nok-1 and Col-0 obtained by sequencing the PCR fragments shown in (A). Differences (SNPs or INDELs) are indicated. The two changes of amino acid are indicated. Polymorphic regions were sequenced at least three times. The positions of the two primers mentioned in (A) are indicated. The PCR amplicon corresponding to Est-1 was sequenced only in regions diverging between Nok-1 and Col-0. The microsatellite polymorphism (MSAT5.08448) used as a genetic marker to genotype the RILs and map the interval (S1 Fig) was identified in both Nok-1 and Col-0, indicating that the amplicons shown in (A) are specific to chromosome 5. Only one transcript is presented for TAD3 (i.e. AT5G24670.2). (PDF) [file pgen.1006551.s002.pdf]

Sup Figure S3

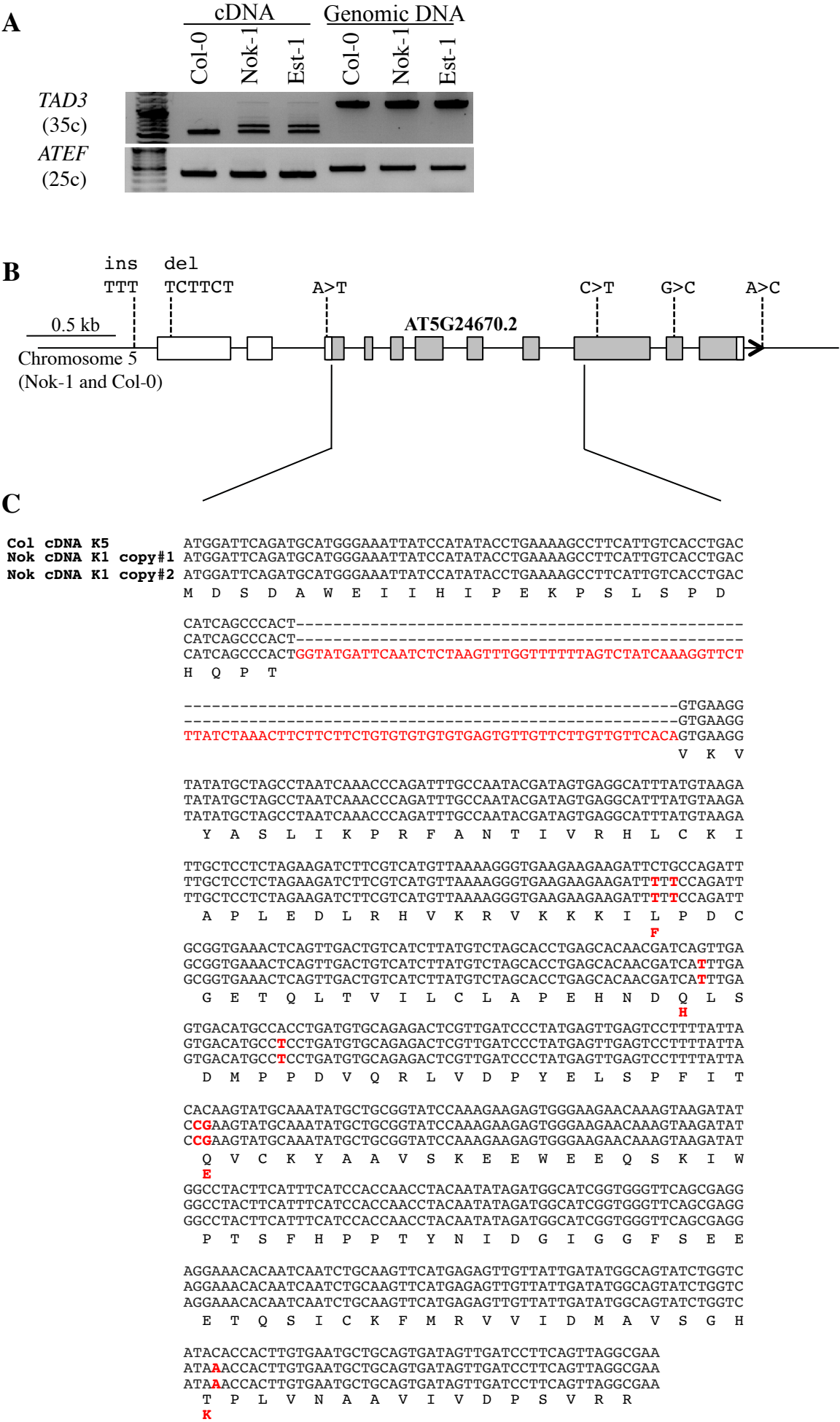

Supplement: S3 Fig — (A) Expression analysis of TAD3 in Col-0, Nok-1 and Est-1. cDNAs were amplified using primers (described in S3 Table) anchored within the coding region. The numbers of PCR cycles are indicated. ATEF cDNA amplifications served as controls. (B) Region amplified corresponding to the PCRs shown in (A). (C) Sequences of the cDNA fragments shown in (A). The upper sequence corresponds to the Col-0 TAD3-1 mRNA. The two other sequences correspond to the TAD3-2 mRNA transcribed from chromosome 1 in Nok-1. Polymorphisms between the sequences are indicated in red: we identified seven SNPs and an insertion of 101 bp corresponding to the retention of one intron. The corresponding amino acids are shown. (PDF) [file pgen.1006551.s003.pdf]

Sup Figure S5

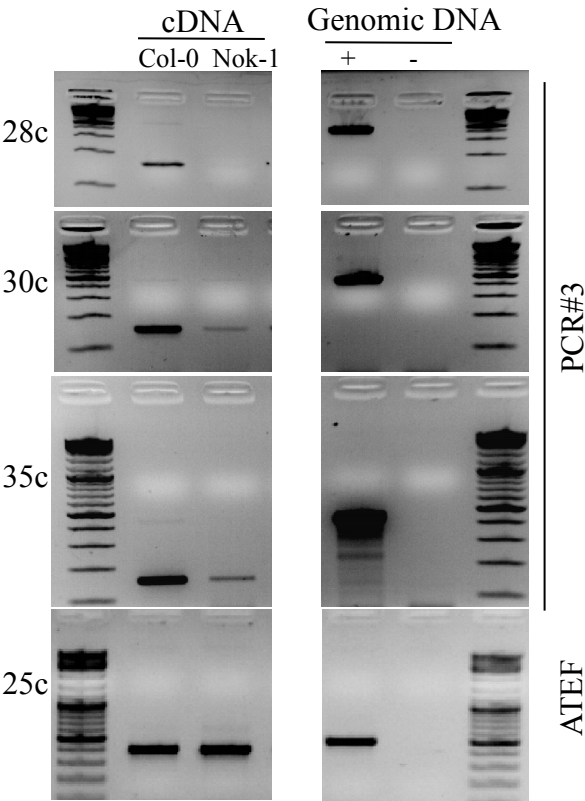

Supplement: S5 Fig — Expression analysis of the TAD3-1 copy in both Nok-1 and Col-0. The region amplified corresponds to PCR#3 (Fig 2A) and is specific for TAD3-1 (S4 Fig). The numbers of cycles are indicated, and Col-0 genomic DNA was used as control. ATEF amplifications served as controls. (PDF) [file pgen.1006551.s005.pdf]

Sup Figure S6

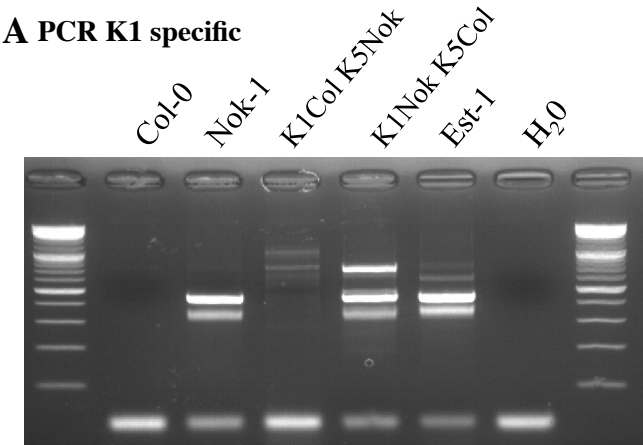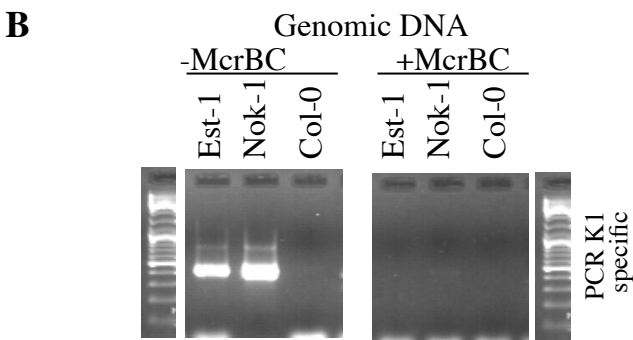

Supplement: S6 Fig — (A) PCR fragments specific for chromosome 1 obtained by amplifying genomic DNAs with primers TAD3_K1Nok Forward and Reverse (S3 Table). The forward primer chromosome 1 specific is designed on a short deletion of 7 bp between chromosomes 1 and 5 (S1 Text). (B) DNA methylation of TAD3 at chromosome 1 analysed by digesting the indicated genomic DNA (300 ng) with McrBC followed by PCR amplification. The region amplified corresponds to the PCR described in (A) and is specific for chromosome 1. (PDF) [file pgen.1006551.s006.pdf]

Sup Figure S7

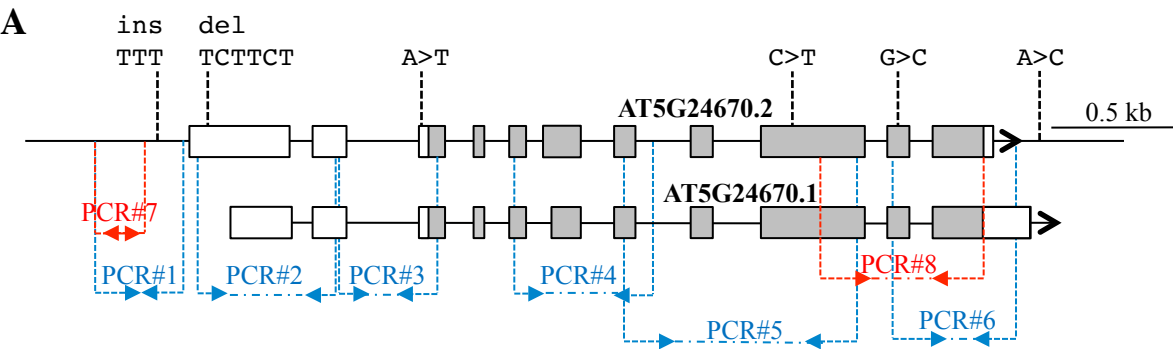

**B PCR#7 (Prom.)**

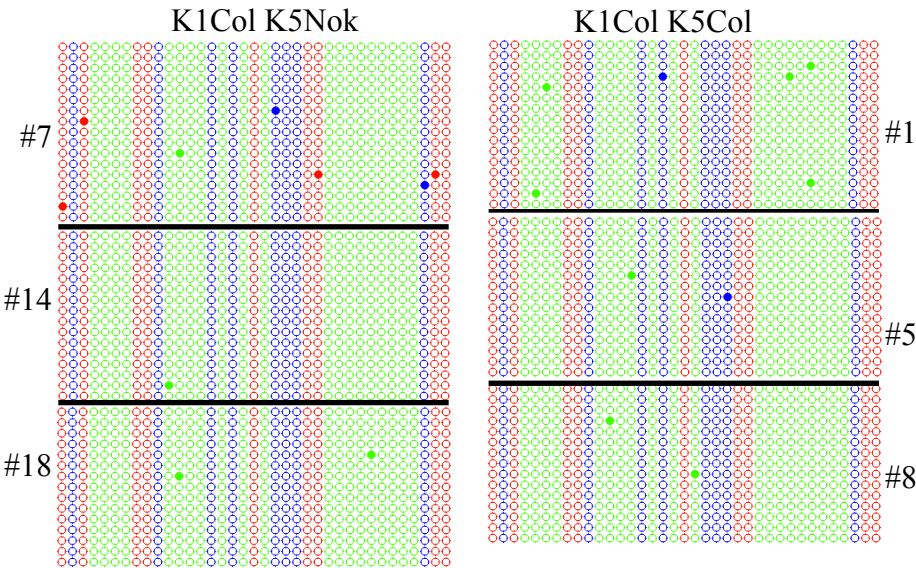

**C PCR#8 (Gene)**

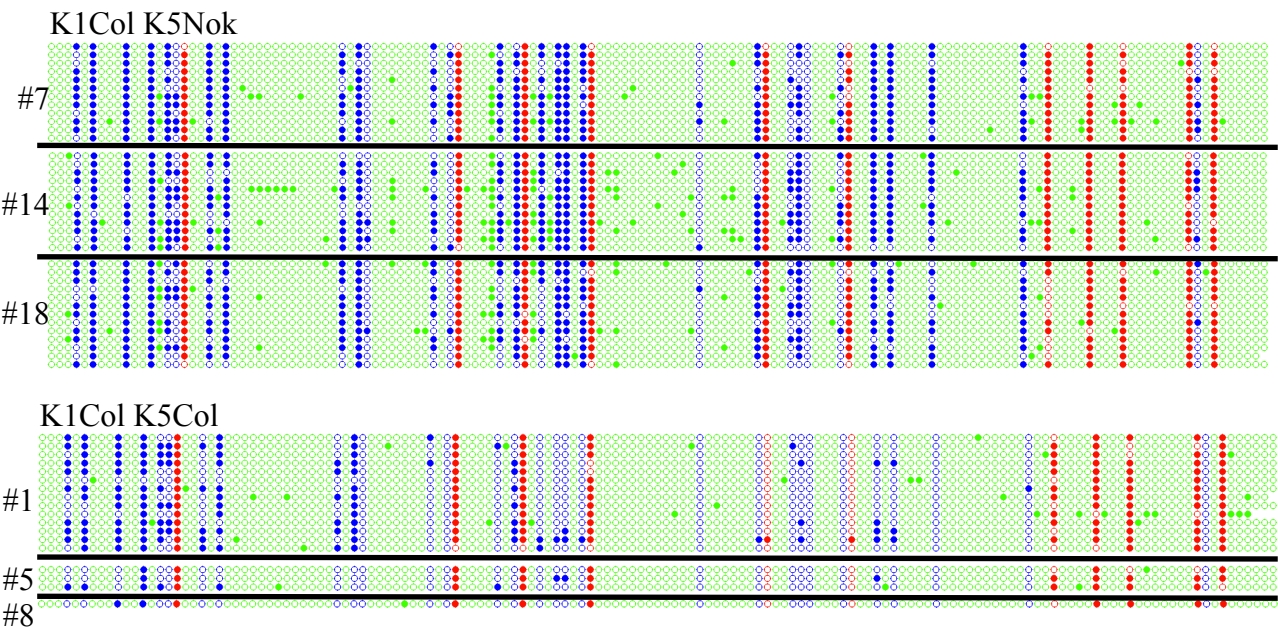

Supplement: S7 Fig — (A) Schematic representation of the TAD3 gene drawn to scale, PCR#7 and PCR#8 amplicons are positioned. (B) and (C) After bisulfite conversion of DNAs, the two regions indicated in (A) were amplified using the primers described in S3 Table. Sequences were aligned using the Kismeth tool [49]. The numbers indicate the plants analyzed, as shown in Fig 4. Cytosines are represented by circles (red: CG, blue: CHG, green: CHH; solid circles: methylated cytosines). (PDF) [file pgen.1006551.s007.pdf]

Sup Figure S8

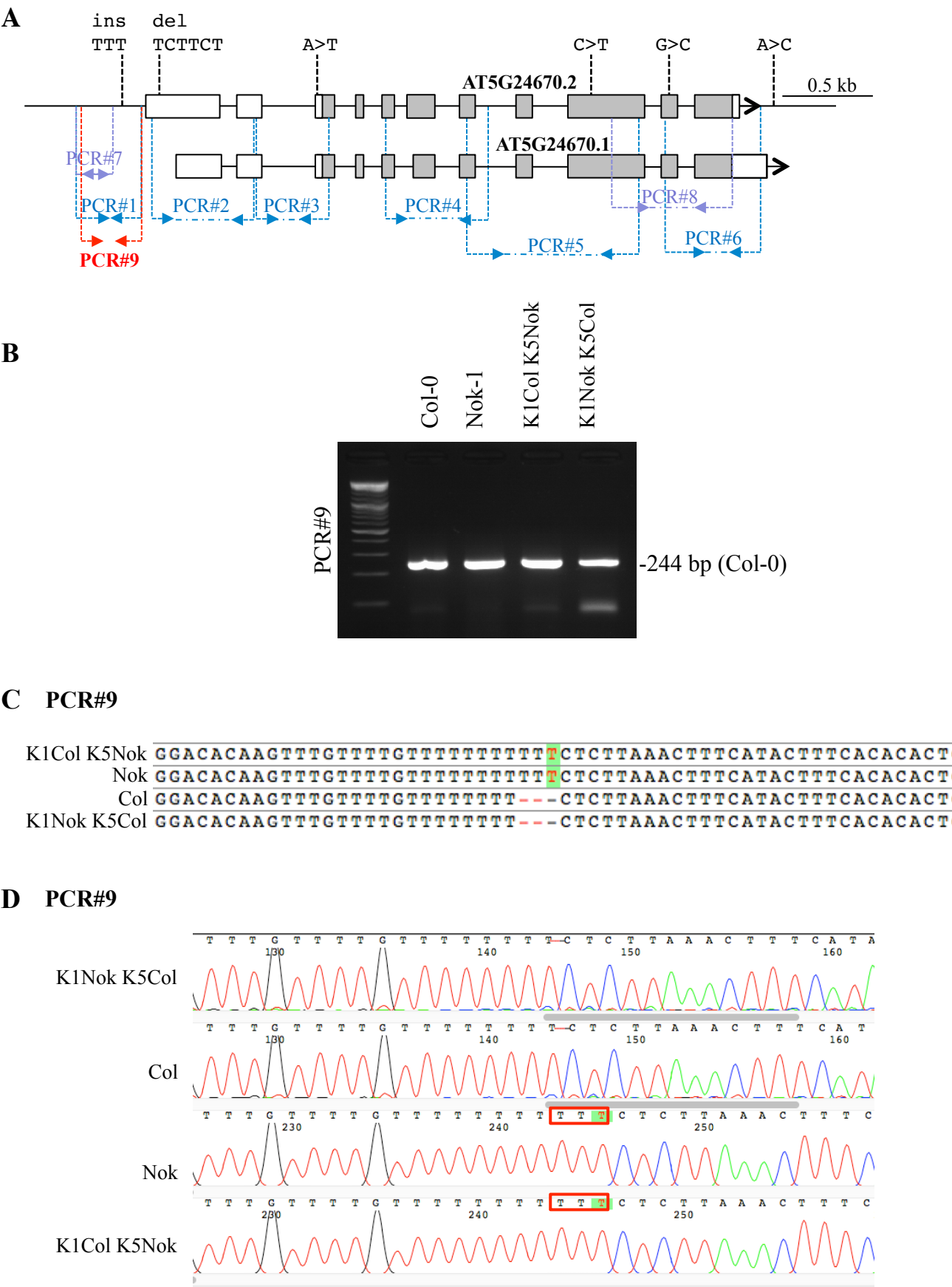

Supplement: S8 Fig — (A) Schematic representation of the TAD3 gene drawn to scale, the PCR#9 amplicon is positioned. (B) PCR amplification on genomic DNAs extracted from plants with the indicated genotypes. K1NokK5Col corresponds to plants from the RIL population that are fixed for the Nok-1 allele at chromosome 1 and for the Col-0 allele at chromosome 5. K1ColK5Nok corresponds to revertant plants from the RIL population that are fixed for the Col-0 allele at chromosome 1 and for the Nok-1 allele at chromosome 5 (see Fig 4). (C) Sequence of the PCR fragments shown in (B). The corresponding electrophoregrams (D) are shown. (PDF) [file pgen.1006551.s008.pdf]

A

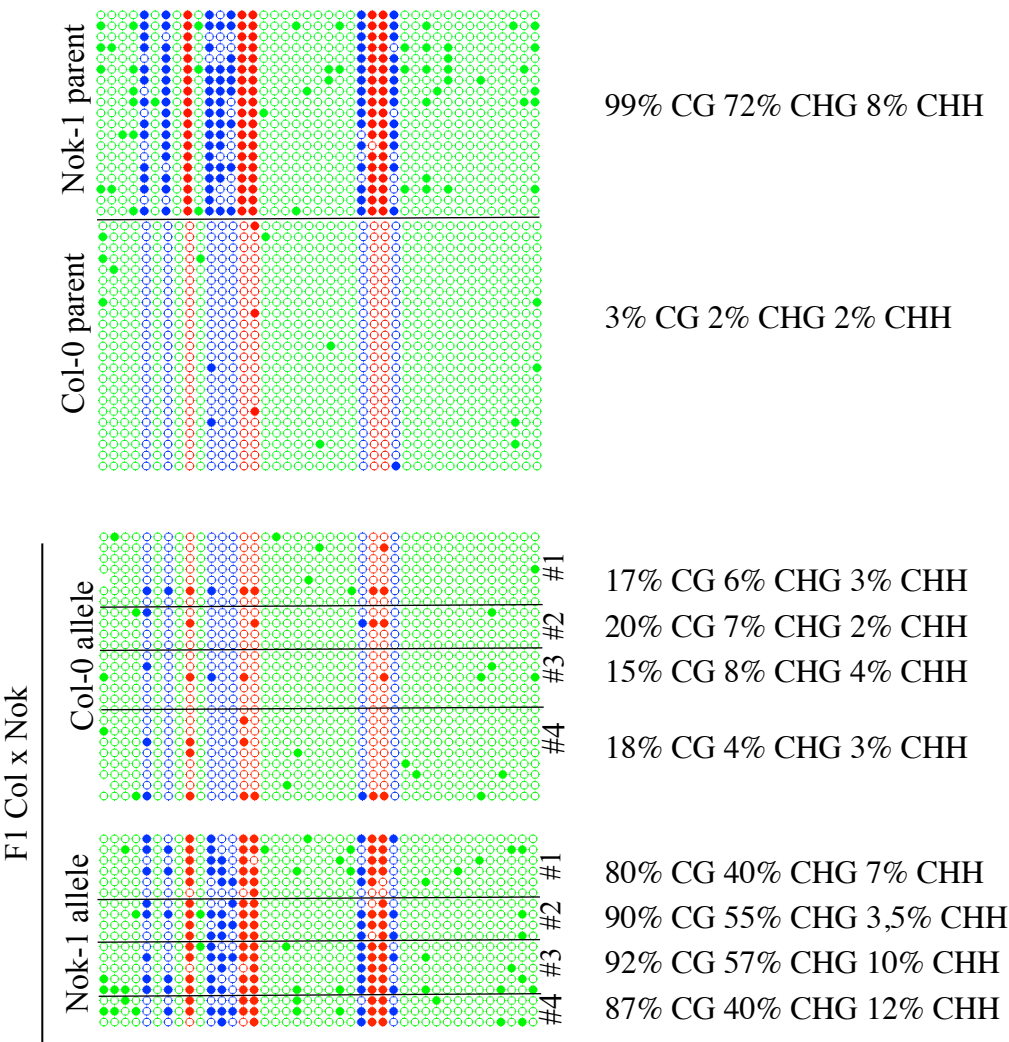

B

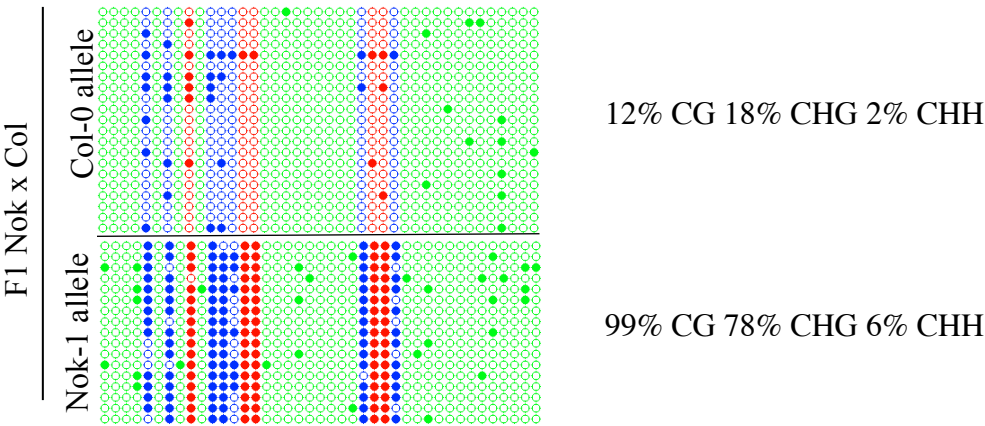

Supplement: S9 Fig — After bisulfite conversion of DNAs, the regions corresponding to PCR#9 (S8 Fig) were amplified using the primers described in S3 Table. Sequences were aligned using the Kismeth tool [49]. (A) Results summarized in Fig 5A with Col-0 and Nok-1 parents and the four individual hybrids resulting from a Col-0 x Nok-1 cross. (B) Methylation patterns obtained in a reciprocal cross. Cytosines are represented by circles (red: CG, blue: CHG, green: CHH; solid circles: methylated cytosines). The amounts of methylated cytosines are indicated in percentages. (PDF) [file pgen.1006551.s009.pdf]

Sup Figure S10

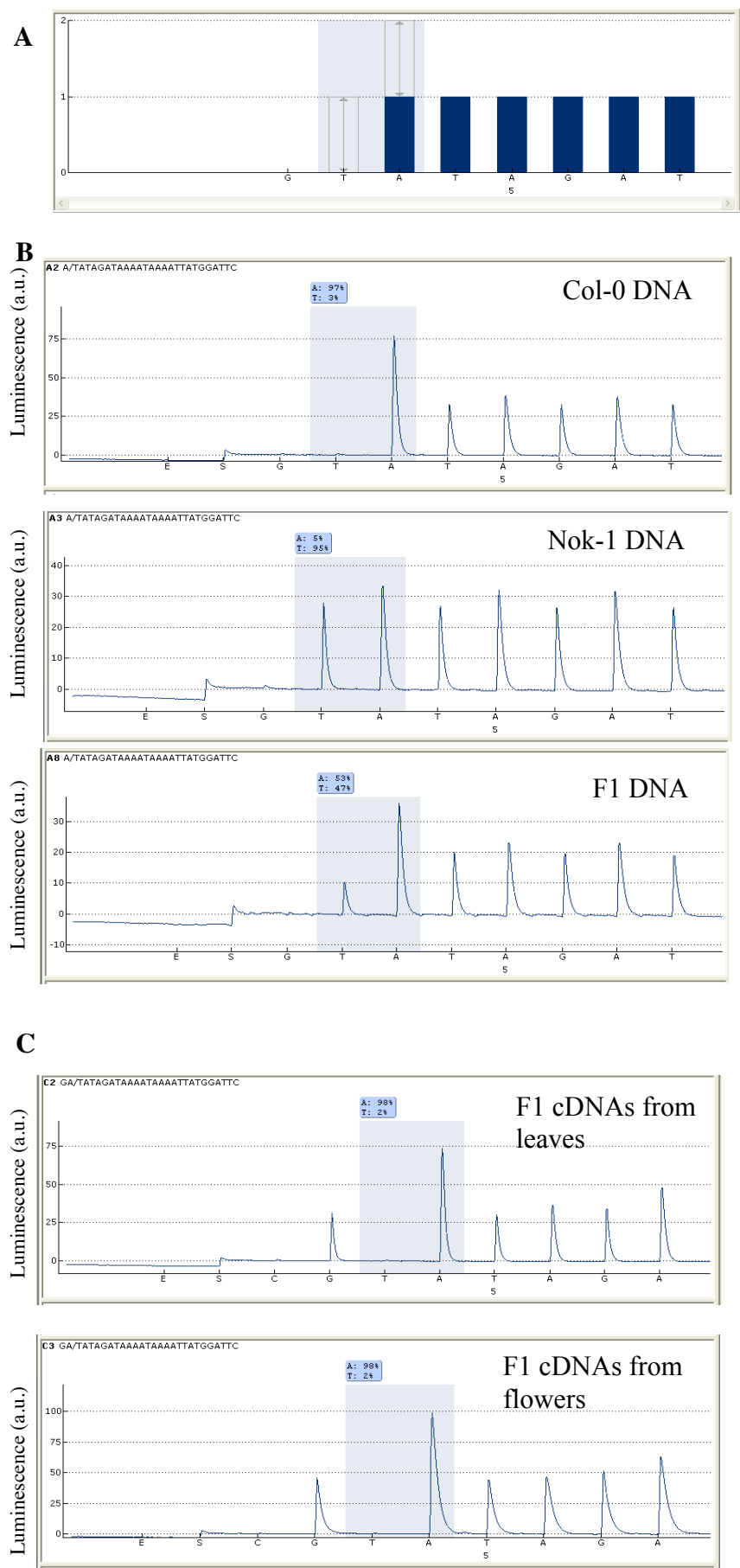

Supplement: S10 Fig — (A) Dispensation order used to analyze the A/T SNP. (B) Pyrograms obtained for the control DNAs. The luminescence is expressed in arbitrary unit (a.u.). Along the abscissa the dispensation order is given with controls (E: enzyme; S: substrate). The SNP position is tinted in grey with the calculated percentage of each nucleotide above. (C) Pyrograms obtained using F1 cDNAs from leaves and flowers. (PDF) [file pgen.1006551.s010.pdf]

Sup Figure S11

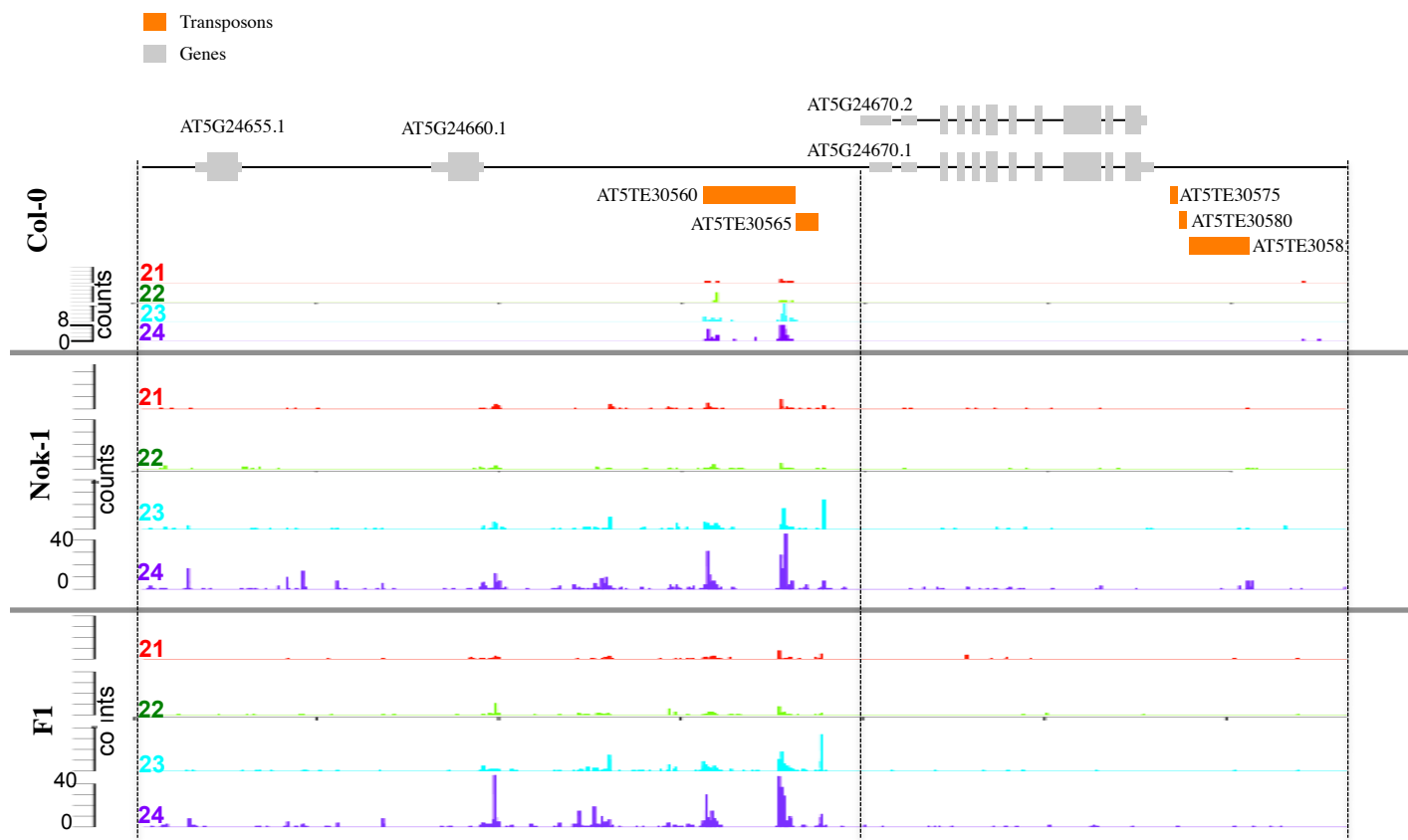

Supplement: S11 Fig — sRNA content of the region corresponding to the mapping interval (Chr5: 8,440,101–8,453,801). sRNAs from Col-0, Nok-1 and the Col-0xNok-1 F1 were mapped to the Col-0 genome (TAIR10.30 version). Both sense and antisense reads were collapsed and only reads corresponding to 21 to 24-nt are plotted. Transposons are in orange. (PDF) [file pgen.1006551.s011.pdf]

Sup Figure S12

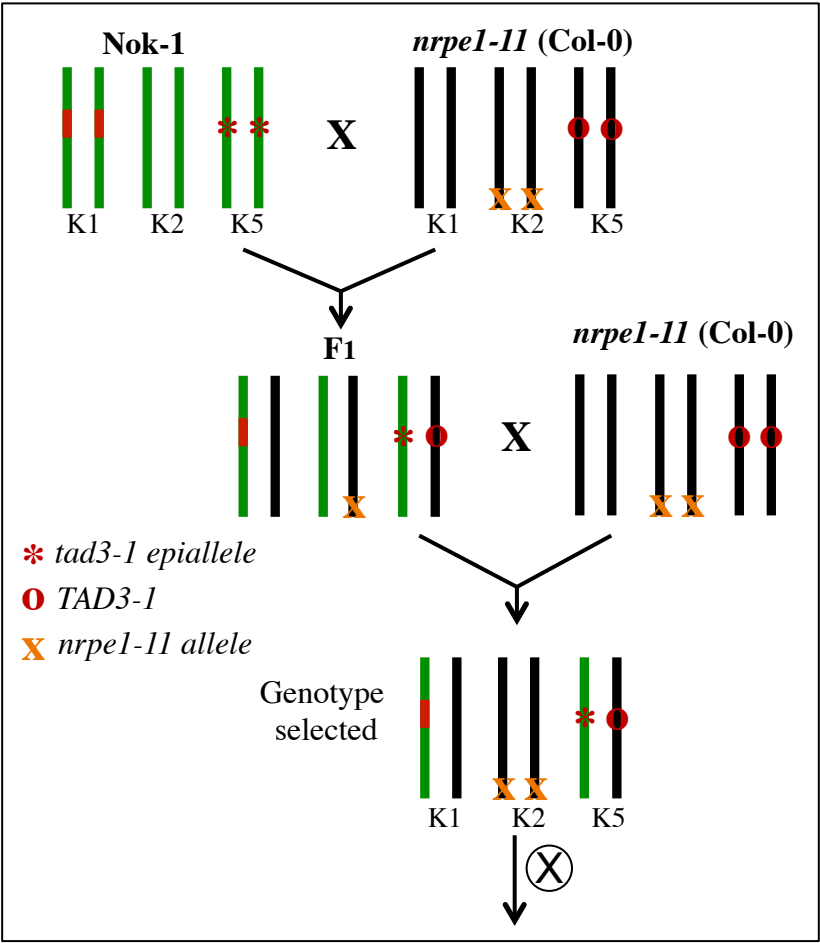

Supplement: S12 Fig — Example of cross between Nok-1 and a mutant (here nrpe1-11) in a Col-0 background performed to obtain a plant fixed for the mutation and heterozygous at both TAD3 loci. (PDF) [file pgen.1006551.s012.pdf]

Sup Figure S13

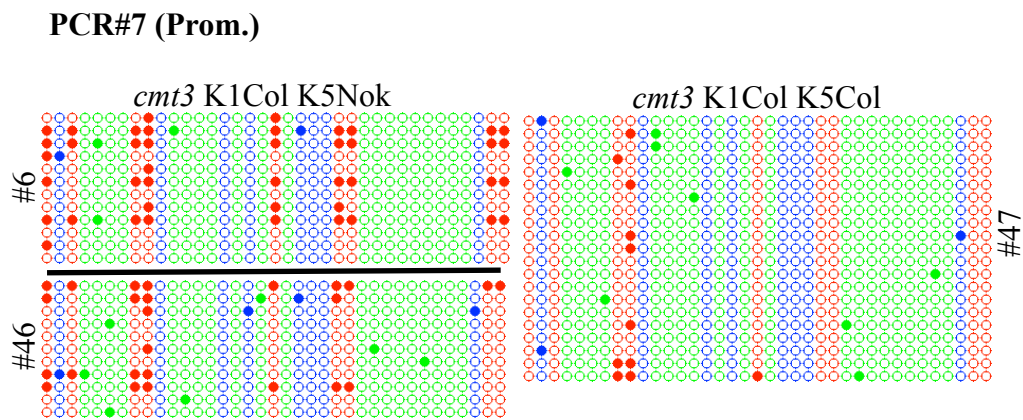

Supplement: S13 Fig — After bisulfite conversion of DNAs, the region corresponding to PCR#7 (S7A Fig) was amplified using the primers described in S3 Table. Sequences were aligned using the Kismeth tool [49]. The numbers indicate the plants analyzed, as shown in Fig 7A. Cytosines are represented by circles (red: CG, blue: CHG, green: CHH; solid circles: methylated cytosines). Results are summarized in Fig 7B. Col-0 and Nok-1 parents are shown in S9A Fig. (PDF) [file pgen.1006551.s013.pdf]
