# Supplementary material for: An Arabidopsis Natural Epiallele Maintained by a Feed-Forward Silencing Loop between Histone and DNA
Source: PLoS Genet. 2017 Jan 6;13(1):e1006551. doi: 10.1371/journal.pgen.1006551 (PMC5257005; doi:10.1371/journal.pgen.1006551)

Sup Figure S4 A-C

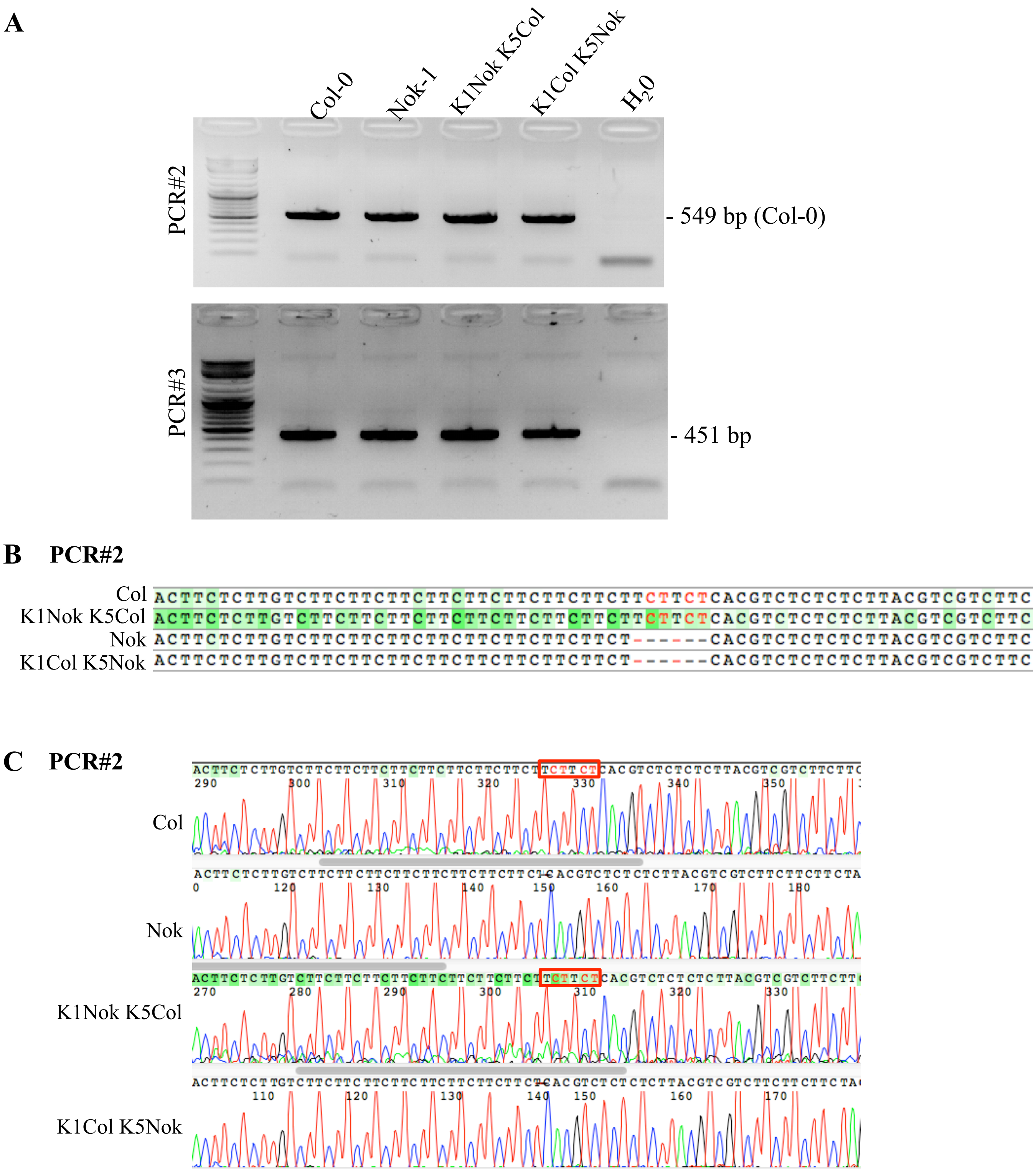

Sup Figure S4 D-E

D **PCR#3**

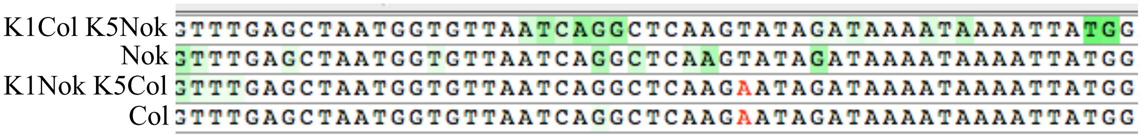

E **PCR#3**

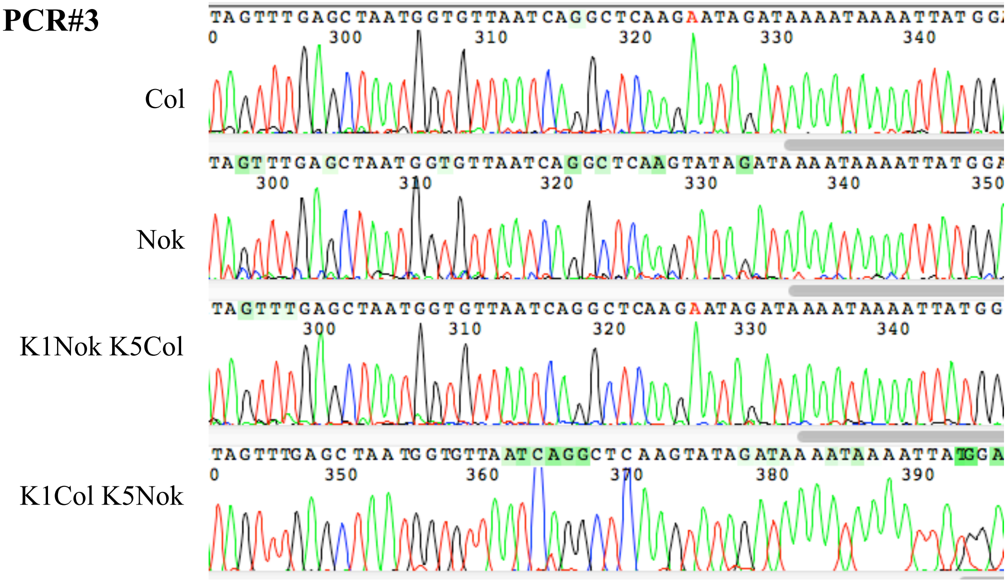

Supplement: S4 Fig — PCR#2 and PCR#3 amplicons are positioned in Fig 2A. The reverse primer for PCR#2 and the forward primer for PCR#3 are overlapping a short deletion of 7 bp between chromosomes 1 and 5 (S1 Text). (A) PCR amplification on genomic DNAs extracted from plants with the indicated genotypes. K1NokK5Col corresponds to plants from the RIL population that are fixed for the Nok-1 allele at chromosome 1 and for the Col-0 allele at chromosome 5. K1ColK5Nok corresponds to revertant plants from the RIL population that are fixed for the Col-0 allele at chromosome 1 and for the Nok-1 allele at chromosome 5 (see Fig 4). (B) and (D) Sequences of the PCR fragments shown in (A). The corresponding electrophoregrams (C) and (E) are shown. (PDF) [file pgen.1006551.s004.pdf]
