# Supplementary material for: An Arabidopsis Natural Epiallele Maintained by a Feed-Forward Silencing Loop between Histone and DNA
Source: PLoS Genet. 2017 Jan 6;13(1):e1006551. doi: 10.1371/journal.pgen.1006551 (PMC5257005; doi:10.1371/journal.pgen.1006551)
Supplement: S1 Table — Using primers (IND5.08449F and IND5.08449R) flanking the deletion of 17 bp (Fig 2), 162 plants from the Nok-1 x Col-0 RIL population were genotyped. The shorter fragment (265 bp) is amplified only when RILs are fixed Nok-1 at chromosome 1, confirming that the deletion is associated with a TAD3 paralog in this region. (PDF) [file pgen.1006551.s014.pdf]

S1 Table: Genotyping of the 17 bp insertion in the Nok-1 x Col-0 RIL population

RIL	IND5.08449F+R	chr1_13869179	chr1_15633764	chr5_07442381	chr5_08563029
11	265 bp +282 bp	Nok	Nok	Col	Col
21	265 bp +282 bp	Nok	Nok	Nok	Nok
27	265 bp +282 bp	Nok	Nok	Col	Col
29	265 bp +282 bp	Nok	Nok	Col	Col
57	265 bp +282 bp	Nok	Nok	Col	Nok
61	265 bp +282 bp	Nok	Nok	Col	Col
64	265 bp +282 bp	Nok	Nok	H	H
66	265 bp +282 bp	Nok	Nok	Col	Col
69	265 bp +282 bp	Nok	Nok	Col	Nok
73	265 bp +282 bp	Nok	Nok	Nok	Nok
76	265 bp +282 bp	Nok	Nok	Col	H
78	265 bp +282 bp	Nok	Nok	Col	Col
81	265 bp +282 bp	H	H	Col	Col
82	265 bp +282 bp	Nok	Nok	Nok	Nok
91	265 bp +282 bp	Nok	Nok	Col	Col
92	265 bp +282 bp	H	H	Nok	Nok
93	265 bp +282 bp	Nok	Nok	Nok	Col
95	265 bp +282 bp	Nok	Nok	Col	Col
96	265 bp +282 bp	Nok	Nok	Nok	Nok
99	265 bp +282 bp	Col	Nok	Col	Col
100	265 bp +282 bp	Nok	Nok	Col	Col
105	265 bp +282 bp	Nok	Nok	Col	Nok
111	265 bp +282 bp	Nok	Nok	Col	Col
112	265 bp +282 bp	Nok	Nok	Col	Col
115	265 bp +282 bp	Nok	Nok	Nok	Nok
123	265 bp +282 bp	Nok	Nok	Nok	Nok
124	265 bp +282 bp	Nok	Nok	Col	Col
130	265 bp +282 bp	Nok	Nok	Nok	Nok
131	265 bp +282 bp	Nok	Nok	Nok	Col
135	265 bp +282 bp	Nok	Nok	Col	Col
140	265 bp +282 bp	Nok	Nok	Nok	Nok
144	265 bp +282 bp	Nok	Nok	Col	Col
148	265 bp +282 bp	Nok	Nok	Nok	Nok
152	265 bp +282 bp	Nok	Nok	Col	Col
153	265 bp +282 bp	Nok	Nok	Col	Col
157	265 bp +282 bp	Nok	Nok	Col	H
158	265 bp +282 bp	Nok	Nok	Col	H
161	265 bp +282 bp	Nok	Nok	Nok	Nok
163	265 bp +282 bp	Nok	Nok	Nok	Nok
166	265 bp +282 bp	Nok	Nok	Col	Nok
169	265 bp +282 bp	Nok	Nok	Nok	Nok
171	265 bp +282 bp	Nok	Nok	Col	Col
174	265 bp +282 bp	Nok	Nok	Nok	Nok
175	265 bp +282 bp	Nok	Nok	Col	Col
179	265 bp +282 bp	Nok	Nok	Nok	Nok
180	265 bp +282 bp	Nok	Nok	Nok	Nok
181	265 bp +282 bp	Nok	Nok	Col	Col
184	265 bp +282 bp	Nok	Nok	Col	Col
187	265 bp +282 bp	Nok	Nok	Col	Nok
188	265 bp +282 bp	Nok	Nok	Nok	Nok
192	265 bp +282 bp	Nok	Nok	Nok	Nok
196	265 bp +282 bp	H	H	Col	Col
197	265 bp +282 bp	Nok	Nok	Col	Col
198	265 bp +282 bp	Nok	Nok	Col	Col
202	265 bp +282 bp	Nok	Nok	Nok	Nok
205	265 bp +282 bp	Nok	Nok	Col	Col
206	265 bp +282 bp	Nok	Nok	Nok	Nok
208	265 bp +282 bp	Nok	Nok	Nok	Nok
209	265 bp +282 bp	Nok	Nok	Nok	Nok
211	265 bp +282 bp	Nok	Nok	Nok	Col
212	265 bp +282 bp	Nok	Nok	Nok	Nok
215	265 bp +282 bp	Nok	Nok	Nok	Nok
217	265 bp +282 bp	H	H	Col	Col
218	265 bp +282 bp	Nok	Nok	Col	Col
219	265 bp +282 bp	Nok	Nok	Nok	Nok
220	265 bp +282 bp	Nok	Nok	Col	Col
226	265 bp +282 bp	Nok	Nok	Col	Col
236	265 bp +282 bp	Nok	Nok	Nok	Nok
239	265 bp +282 bp	Nok	Nok	Col	Col
248	265 bp +282 bp	Nok	Nok	Col	Col
255	265 bp +282 bp	Nok	Nok	Col	Col
261	265 bp +282 bp	Nok	Nok	Nok	Nok
262	265 bp +282 bp	Nok	Nok	Nok	Nok
267	265 bp +282 bp	Nok	Nok	Col	Col
270	265 bp +282 bp	Nok	Nok	Nok	Nok
271	265 bp +282 bp	Nok	Nok	Col	Col
277	265 bp +282 bp	Nok	Nok	Nok	Nok
278	265 bp +282 bp	Nok	Nok	Nok	Nok
279	265 bp +282 bp	Nok	Nok	Nok	Nok

281	265 bp +282 bp	Nok	Nok	Nok	Nok
282	265 bp +282 bp	Nok	Col	Nok	Nok
286	265 bp +282 bp	Nok	Nok	Col	Col
289	265 bp +282 bp	Nok	Nok	Nok	Nok
293	265 bp +282 bp	Nok	Nok	Nok	Nok
301	265 bp +282 bp	Nok	Nok	Nok	Col
304	265 bp +282 bp	Nok	Nok	Nok	Nok
309	265 bp +282 bp	Nok	Nok	Nok	Col
312	265 bp +282 bp	Nok	Nok	Nok	Nok
326	265 bp +282 bp	Nok	Nok	H	H
327	265 bp +282 bp	Nok	Nok	Col	Col
334	265 bp +282 bp	Nok	Nok	Col	Col
340	265 bp +282 bp	Nok	Nok	Nok	Nok
353	265 bp +282 bp	Nok	Nok	Col	Col
359	265 bp +282 bp	Nok	Nok	Col	Col
363	265 bp +282 bp	Nok	Nok	Nok	Nok
369	265 bp +282 bp	Nok	Nok	Nok	Nok
370	265 bp +282 bp	Nok	Nok	Nok	Nok
371	265 bp +282 bp	Nok	Nok	Col	Nok
372	265 bp +282 bp	Nok	Nok	Nok	Nok
374	265 bp +282 bp	Nok	Nok	Nok	Nok
377	265 bp +282 bp	Nok	Nok	Nok	Nok
380	265 bp +282 bp	Nok	Nok	Col	Col
381	265 bp +282 bp	Nok	Nok	Nok	Col
382	265 bp +282 bp	Nok	Nok	Nok	Col
383	265 bp +282 bp	Nok	Nok	Col	Col
384	265 bp +282 bp	Nok	Nok	Col	Nok
386	265 bp +282 bp	Nok	Nok	Col	Col
387	265 bp +282 bp	Nok	Nok	Nok	Nok
394	265 bp +282 bp	Nok	Nok	Nok	Nok
395	265 bp +282 bp	Nok	Nok	Col	Col
396	265 bp +282 bp	Nok	Nok	Nok	Nok
401	265 bp +282 bp	Nok	Nok	Col	Col
405	265 bp +282 bp	Nok	Nok	Col	Col
406	265 bp +282 bp	Nok	Nok	Nok	Nok
409	265 bp +282 bp	Nok	Nok	Col	Col
410	265 bp +282 bp	H	H	Col	Col
411	265 bp +282 bp	Nok	Nok	Nok	Nok
414	265 bp +282 bp	Nok	Nok	Nok	Nok
416	265 bp +282 bp	Nok	Nok	Col	Col
417	265 bp +282 bp	Nok	Nok	Nok	Nok
418	265 bp +282 bp	Nok	Nok	Col	Nok
419	265 bp +282 bp	Nok	Nok	Col	Col
420	265 bp +282 bp	Nok	Nok	Nok	Nok
422	265 bp +282 bp	Nok	Nok	Col	Col
427	265 bp +282 bp	Nok	Nok	Col	Col
5	282 bp	Col	Col	Col	Col
26	282 bp	Col	Col	H	H
35	282 bp	Col	Col	Col	Col
45	282 bp	Col	Col	Col	Col
98	282 bp	Col	Col	Col	Col
101	282 bp	Col	Col	Col	Col
104	282 bp	Col	Col	H	H
113	282 bp	Col	Col	Nok	Col
114	282 bp	Col	Col	Col	Col
154	282 bp	Col	Col	H	Col
156	282 bp	Col	Col	Col	Col
177	282 bp	Col	Col	Nok	Col
182	282 bp	Col	Col	Col	Col
185	282 bp	Col	Col	Col	Col
190	282 bp	Col	Col	Col	Col
191	282 bp	Col	Col	Col	Col
207	282 bp	Col	Col	Col	H
227	282 bp	Col	Col	Nok	Col
228	282 bp	Col	Col	Nok	Col
231	282 bp	Col	Col	Col	Col
235	282 bp	Col	Col	Nok	H
238	282 bp	Col	Col	Col	Col
245	282 bp	Col	Col	Nok	Col
253	282 bp	Col	Col	Col	Col
257	282 bp	Col	Col	Col	Col
266	282 bp	Col	Col	Col	Col
349	282 bp	Col	Col	Col	Col
350	282 bp	Col	Col	Nok	Col
355	282 bp	Col	Col	H	H
356	282 bp	Col	Col	Col	Col
366	282 bp	Col	Col	Col	Col
375	282 bp	Col	Col	Col	Col
385	282 bp	Col	Col	Col	Col
391	282 bp	Col	Col	Col	Col
415	282 bp	Col	Col	H	H
424	282 bp	Col	Col	Col	Col
426	282 bp	Col	Col	Col	Col
