# Supplementary material for: An Arabidopsis Natural Epiallele Maintained by a Feed-Forward Silencing Loop between Histone and DNA
Source: PLoS Genet. 2017 Jan 6;13(1):e1006551. doi: 10.1371/journal.pgen.1006551 (PMC5257005; doi:10.1371/journal.pgen.1006551)
Supplement: S2 Table — (PDF) [file pgen.1006551.s015.pdf]

S2 Table: Small RNA seq statistics

	Col-0	Nok-1	F1 Col x Nok
Total reads processed:	9 819 426	15 866 240	21 830 096
Reads with adapters:	9 514 345	15 336 830	21 012 571
Reads that were too short:	2 914 985	4 054 998	6 466 951
Reads written (>15nt):	6 904 441	11 811 242	15 363 145
Unique mappers:	884 546	2 053 107	2 556 375
Multi mappers:	5 264 866	8 718 639	11 471 372
Multi mappers ignored and marked as unmapped:	46 084	62 709	94 440
Non mapper:	708 945	976 787	1 240 958
Total mapped	6 149 412	10 771 746	14 027 747
	63%	68%	64%
