# Supplementary material for: An Arabidopsis Natural Epiallele Maintained by a Feed-Forward Silencing Loop between Histone and DNA
Source: PLoS Genet. 2017 Jan 6;13(1):e1006551. doi: 10.1371/journal.pgen.1006551 (PMC5257005; doi:10.1371/journal.pgen.1006551)
Supplement: S3 Table — (PDF) [file pgen.1006551.s016.pdf]

S3 Table: Primers

Primer name	5'-3' sequence
Ind5.08448FNok	GAGTCTTTATTATCATCTAAGC
TAD3F	CTCGATACAGTAGCTGCTCC
TAD3R	TATGAACACTCCCGAGACCAC
TAD3_K1_F	GAGTCTTTATTATCATCTAAGC
TAD3_K1_R	GCTTAGATGATAATAAAGACTC
Pyro_seq	CTCTCCAGCGCTCAA
SEQ5.08447F	TTGGGGTTTAATGGTTTTCG
SEQ508451R_3UTR3	CTCTAAAACACAAGTTTCTGACTAG
IND5.08449F	GAGTGACATGCCACCTGATG
IND5.08449R	TGTGTTTCCTCCTCGCTGA
SEQ5.08447F	TTGGGGTTTAATGGTTTTCG
SEQ508451R_3UTR3	CTCTAAAACACAAGTTTCTGACTAG

RT-PCR (S3A Fig)

Gene	Primer set	5'-3' sequence
TAD3-1 AT5G24670	Seq5.08448F	AAATTATGGATTCAGATGCA
	SEQ5.08449R	TTCGCCTAACTGAAGGATCAA
AtEf1alpha	Ef1alpha f	GCACTGTCATTGATGCTCC
	Ef1alpha r	GTCAAGAGCCTCAAGGAGAG

PCR fragments	Primer forward (5'-3' sequence)	Primer reverse (5'-3' sequence)
PCR#1	ATYTGATTYATAGGATTGGGGTTTAATGGTT	AAATCTCCTATATATATGTGTAATGTGTGTAAACTTAT
PCR#2	AGAATAAGCTACTTCTACGAATGG	CGCAGCGAGCGATAGCCG
PCR#3	CTTCCTCTCTATCGGCTATCGC	TGGTCAGGTGACAATGAAGG
PCR#4	GAGGTTTTTATTGTAAGCTTATTGGACAGGTG	AACGACTAAAGCATATATAATAAAAAGACCA
PCR#5	AGAAGAGTGGGAAGAAYAAAGTAAG	AACATRATCCTRATCAAAAATCTT
PCR#6	TGAGTTATYGATAGGTYYAAAATGAYAGYAGAGAAGAGA	CAAARAATTARCTTTCTCACCCARAACCTRATTATCAATC
PCR#7	ATYTGATTYATAGGATTGGGGTTTAATGGTT	CAAAACAAACTTRTRTCCTAARCARAAAAACAACATC
PCR#8	GAGGAAAYAGGGGAAATATGTTTAA	CAAAACTCTAAACACTRCATAAT
PCR#9	TAAACGTGGGTTTCGAAAAATTGAATTCTGTGTAG	AAATCTCCTATATATATGTGTAATGTGTGTAAACTTAT

Markers presented in S1 Fig:

Marker name	Polymorphism	Primer forward (5'-3' seq)	Primer reverse (5'-3' seq)
MSAT1.11723	microsatellite	GCAAGCATGCCTTCAACTTT	TTTCGTTTTTCTGGTCCACAC
MSAT1.13303	microsatellite	GGATCATGAATCTCCATTCTCTG	CTCTTTTTGATGTTTGGACCAT
MSAT1.14160	microsatellite	CTAAACTAGAACCAGGGGTAA	ACAAAAATCGTGGTGATAATA
MSAT1.15597	microsatellite	GTCCCTGCACGTTTTAGGTC	CACATGGTTTTGCTCCCAAT
MSAT1.15896	microsatellite	ACCTAACAGCGACCGTCAGT	ACCAACCTGAAAACGTGAGG
MSAT1.16510	microsatellite	AACCTTTCTCGTTGATTTCCAA	CCAAGCAAGAAGGCAAAATC
MSAT5.07067	microsatellite	TGTATCTGAAGTAGCACGAC	TTTAAGCAGGTGAGAATTGTA
MSAT5.07870	microsatellite	TAATGCCATCTTGCAGGTGA	AAATTTTGTTGACCCCCAAT
MSAT5.08049	microsatellite	CCGTCCAAGCAAACTACACA	TGCGGTGAGTTTCACTGTTT
MSAT5.08122	microsatellite	TCGCTGTTTCAAAAAGTCGTAG	GCACATAGCACCACACCATC
MSAT5.08428	microsatellite	CTCGGGTCAAAATTAGGGTTTCG	GCTACCAGATCCGATGGTAAGATG
SEQ5.08440	SNP	GGAGAAGGAGCCTAATTGCAT	TCGGAGGTAGAGGAGCTACG
SEQ5.08453	SNP	GCTTTGTGTCCTGTCTGCAA	CGTCTCTGAACCCCAACAAT
SEQ5.08464	SNP	GGGATAGCACGAGGATCAAA	TGACAAGGCCGTTATGTGAA
MSAT5.08611	microsatellite	GAAAAGCAGGCTACGTCAAAA	ACCGCGTAAAGTTCATGTCC
MSAT5.09007	microsatellite	CCCAATACATGCATGAAGAGAA	CGGTCTTGTAGATTGATCAGG
MSAT5.09792	microsatellite	TTGCTTTTTGGTTATATTCGGA	ATCATCTGCCCATGGTTTTT

The marker names give the number of the chromosome followed by the physical position in kb of the markers on the chromosome (TAIR 10.0 Col-0 genomic sequence, http://www.arabidopsis.org)
