# Supplementary material for: An Arabidopsis Natural Epiallele Maintained by a Feed-Forward Silencing Loop between Histone and DNA
Source: PLoS Genet. 2017 Jan 6;13(1):e1006551. doi: 10.1371/journal.pgen.1006551 (PMC5257005; doi:10.1371/journal.pgen.1006551)
Supplement: S1 Text — SNPs/indels found between chromosomes 1 and 5 are in red. SNPs/indels found between the copies mapped at chromosome 1 are in blue. Stars indicate nucleotides that are identical between all sequences. Bold stars and sequences underlined indicate transcribed regions. Start and stop codons are in bold. The SNPs identified in the cDNAs (S3 Fig) correspond to TAD3-2. The deletion of 7 bp (ATCGGCT) between the copies of chromosomes 1 and 5 was used to design primers specific for chromosome 1 (Fig 3A). The reverse primer for PCR#2 and the forward primer for PCR#3 are overlapping this deletion, and are specific for chromosome 5. (PDF) [file pgen.1006551.s017.pdf]

```
Nok1 K5 TAD3-1  CTCGATACAGTAGCTGCTCCGCTCATCAACGTCCATTTCGCCGCCTTTAGCCGTCAACGT
Nok1 K1 TAD3-2  CTCGATACAGTAGCTGCTCCGCTCATCAACGTCCATTTCGCCGCCTTTAGCCGTCAACGT
Nok1 K1 TAD3-3  CTCGATACAGTAGCTGCTCCGCTCATCAACGTCCATTTCGCCGCCTTTAGCCATCAACGT
                **************************************************** *******

Nok1 K5 TAD3-1  TCTCTGGTTCTGTTGCCGTACAAAATTCTTAAAGGTCGCGTTTTTATGTAAGCGTAGGGT
Nok1 K1 TAD3-2  TCTCTGGTTCTGTTGCCGTACAAAATTCTTAAAGGTCGCGTTTTTATGTAAGCGTAGGGT
Nok1 K1 TAD3-3  TTTCTGGTTCTGTTGTAGTACAAAATTCTTAAAGGTCGCGTTTTTATGTAAGCGTAGGGT
                * *************  *******************************************

Nok1 K5 TAD3-1  TTAGTTGTCGTCCCGCTCTTTTTAATTAGACTTTGTTAATTTAATTAGGTTAATTAGATA
Nok1 K1 TAD3-2  TTAGTTGTCGTCCCGCTCTTTTTAATTAGACTTTGTTAATTTAATTAGGTTAATTAAATA
Nok1 K1 TAD3-3  TTAGTTGTCGTCCCGCTCCTTTTAATTAGACTTTGTTAATTTAATTAGGTTAATTAGATA
                ****************** ************************************** ***

Nok1 K5 TAD3-1  AGACCTAAAACGGCGACGTGTAAGCTGTCGTCTTCTTGACCTTTTTGGTGAACTCCTCCT
Nok1 K1 TAD3-2  AGACCTAAAACAGCGACGTGTAAGCTGTCGTCTTCTCGACCTTTTTGGTGAACTCCTCCT
Nok1 K1 TAD3-3  AGACCTAAAACGGCGACGTGTAAGCTGTCGTCTTCTTGACCTTTTTGGTGAACTCCTCCT
                *********** ************************ ***********************

Nok1 K5 TAD3-1  TCCTCTCTATCGGCTATCGCTCGCTGCGTCTCTGTGTCTTGTTGCTTGGAGCAAGCTCTC
Nok1 K1 TAD3-2  TCTTCTCT-------ATAGCTCGTTGCGTCTCTGTGTCTTGTTTCTTGGAGCAAGCTCTC
Nok1 K1 TAD3-3  TCTTCTCT-------ATAGCTCGCTGCGTCTCTGTGTCTTGTTGCTTGGAGCAAGCTCTC
                ** *****       ** ***** ******************* ****************

Nok1 K5 TAD3-1  CAGCGTGAGTTAATCTAATCTCTGTAATGATTAATTACCTTCTTTTACGGTTTCTGGAAT
Nok1 K1 TAD3-2  CAGTGTGAGTTAATCTAATCTCTGTAATGATTAATTACCTTCTTTTACGGTTTCTGAAAT
Nok1 K1 TAD3-3  CAGCGTGAGTTAATCTAATCTCTGTAATGATTAATCACCTTCTTTTACGGTTTCTGGAAT
                *** ******************************* ******************** ***

Nok1 K5 TAD3-1  TGCATCTGTGTGTGAGCACTTGGTTTGAAATATGATTTAACTAGTGAATTCGATAATGCT
Nok1 K1 TAD3-2  TGCATCTATGTGTGAGCACTTGGTTTGAAATATGATTTAACTAGTGAATTCGATAATGCT
Nok1 K1 TAD3-3  TGCATCTGTGTGTGAGCACTTGGTTTAAAAAATGATTTAACTAGTGAATTCGATAATGCT
                ******* ****************** *** *****************************

Nok1 K5 TAD3-1  GTTCAATAAGAATGCTTAGAAAAGCCTTAACTGGGTTTTACTCACCACTCTTCTTATTGA
Nok1 K1 TAD3-2  GTTCAATAAGAATGCTTAGAAAAGCCTTAACTGAGTTTTATTTACCACTCTTCTTATTGA
Nok1 K1 TAD3-3  GTTCAATAAGAATGCTTAGAAAAGCCTTAACTGAGTTTTACTCACCACTCTTCTTATTGA
                ********************************* ****** * *****************

Nok1 K5 TAD3-1  ATCTAGAAATTGAAAGTGTGAATTTTTGCGAGTTCTCTTATTCAAACTTCACTGCGAAAG
Nok1 K1 TAD3-2  ATCTAGAAATTGAAAGTGTGAATTTTTGCGAGTTCTCTTATTCAAACTCCACTGAGAAAG
Nok1 K1 TAD3-3  ATCTAGAAATTGAAAGTGTGAATTTTTGCGAGTTCTCTTATTCAAACTCCACTGAGAAAG
                ************************************************ ***** *****

Nok1 K5 TAD3-1  GTGGAGTTTTTCGTCTCTGTTTTCTCCCTAATTCTTATAGTTTGAGCTAATGGTGTTAAT
Nok1 K1 TAD3-2  GTGGAGTTTTTCGTCTCTGTTTTCTCCCTAATTCTTATAGTTTGAGCTAATGGTGTTAAT
Nok1 K1 TAD3-3  GTGGAGTGTTTCGTCTCTGTTTTCTCCCTAATTCTTATAGTTTGAGCTAATGGTGTTAAT
                ******* ****************************************************

Nok1 K5 TAD3-1  CAGGCTCAAGTATAGATAAAATAAAATTATGGATTCAGATGCATGGGAAATTATCCATAT
Nok1 K1 TAD3-2  CAGGCTCAAGTATAGATAAAATAAAATTATGGATTCAGATGCATGGGAAATTATCCATAT
Nok1 K1 TAD3-3  CAGGCTCAAGTATAGATAAAATAAAATTATGGATTCAGATGCATGGGAAATTATCCATAT
                ************************************************************

Nok1 K5 TAD3-1  ACCTGAAAAGCCTTCATTGTCACCTGACCATCAGCCCACTGGTATGATTCAATCTCTAAG
Nok1 K1 TAD3-2  ACCTGAAAAGCCTTCATTGTCACCTGACCATCAGCCCACTGGTATGATTCAATCTCTAAG
Nok1 K1 TAD3-3  ACCTGAAAAGCCTTCATTGTCACCTGACCATCAGCCCACTGATATGATTCAATCTCTAAG
                ***************************************** ******************

Nok1 K5 TAD3-1  TTTGGTTTTTTAGTCTATCAAAGGTTCTTTATCTAAACTTCTTCTTCTGTGTGT--GTGT
Nok1 K1 TAD3-2  TTTGGTTTTTTAGTCTATCAAAGGTTCTTTATCTAAACTTCTTCTTCTGTGTGT--GTGT
Nok1 K1 TAD3-3  TTTGGTTTTTTAGTCTATCAAAGGTTCTTTATCTAAACTTCTTCTTCTGTGTGTGTGTGT
                ******************************************************  ****

Nok1 K5 TAD3-1  GTGTGTTGTTCTTGTTGTTCACAGTGAAGGTATATGCTAGCCTAATCAAACCCAGATTTG
Nok1 K1 TAD3-2  GAGTGTTGTTCTTGTTGTTCACAGTGAAGGTATATGCTAGCCTAATCAAACCCAGATTTG
Nok1 K1 TAD3-3  GAGTGTTGTTCTTGTTGTTCACAGTGAAGGTATATGCTAGCCTAATCAAACCCAGATTTG
                * **********************************************************

Nok1 K5 TAD3-1  CCAATACGATAGTGAGGTTTGTCATTCTTGTTTCGACTCTTGACTCGTTGATTCCAGTGC
Nok1 K1 TAD3-2  CCAATACGATAGTGAGGTTTGTCATTCTTGTTTCGACTCTTGACTCGTTGATTTCAGTGC
Nok1 K1 TAD3-3  CCAATACGATAGTGAGGTTTGTCATTCTTGTTTCGACTCTTGACTCGTTGATTTCAGTGC
                ***************************************************** ******
```

```
Nok1 K5 TAD3-1  TGTAATCTCATGAGTGAATTTTTTTACTTTGCTATGATAGGCATTTATGTAAGATTGCTC
Nok1 K1 TAD3-2  TGTAATCTCATGAGTGAATTTTTTGACTTTGCTATGATAGGCATTTATGTAAGATTGCTC
Nok1 K1 TAD3-3  TGTAATCTCATGAGTGAATTTTTTGACTTTGCTATGATAGGCATTTATGTAAGATTGCTC
                ************************ ***********************************

Nok1 K5 TAD3-1  CTCTAGAAGATCTTCGTCATGTTAAAAGGGTGAAGAAGAAGATTCTGCCAGATTGCGGTA
Nok1 K1 TAD3-2  CTCTAGAAGATCTTCGTCATGTTAAAAGGGTGAAGAAGAAGATTTTTCCAGATTGCGGTA
Nok1 K1 TAD3-3  CTCTAGAAGATCTTCGTCATGTTAAAAGGGTGAAGAAGAAGATTCTTCCAGATTGCGGTA
                ******************************************** * *************

Nok1 K5 TAD3-1  TCCCATTGTTAGTTTTCTTAGCGCGCTGATACACCAATCATAAGATTATGAGGTTTTTAT
Nok1 K1 TAD3-2  TCCCATTGTTAGTTTTCTTAGCGCGCTGATACACCAATCATAAGATTATGAGGTTTTTAT
Nok1 K1 TAD3-3  TCCCATTGTTAGTTTTCTTGGTGCGCTGATACACCAATCATAAGATTATGAGGTTTTTAT
                ******************* * **************************************

Nok1 K5 TAD3-1  TGTAAGCTTATTGGACAGGTGAAACTCAGTTGACTGTCATCTTATGTCTAGCACCTGAGC
Nok1 K1 TAD3-2  TGTAAGCTTATTGGACAGGTGAAACTCAGTTGACTGTCATCTTATGTCTAGCACCTGAGC
Nok1 K1 TAD3-3  TGTAAGCTTATTGGACAGGTGAAACTCAGTTGACTGTCATCTTATGTCTAGCACCTGAGC
                ************************************************************

Nok1 K5 TAD3-1  ACAACGATCAGTTGAGTGACATGCCACCTGATGTGCAGAGACTCGTTGATCCCTATGAGT
Nok1 K1 TAD3-2  ACAACGATCATTTGAGTGACATGCCTCCTGATGTGCAGAGACTCGTTGATCCCTATGAGT
Nok1 K1 TAD3-3  ACAACGATCATTTGAGTGACATGCCTCCTGATGTGCAGAGACTCGTTGATCCCTATGAGT
                ********** ************** **********************************

Nok1 K5 TAD3-1  TGAGTCCTTTTATTACACAAGTAGGT-TATTTTTCTCTTATCTCACCACTATTTGCTTCA
Nok1 K1 TAD3-2  TGAGTCCTTTTATTACCGAAGTAGGTTTATTTTTCTCTTTTCTCACCACTATTTGCTTCA
Nok1 K1 TAD3-3  TGAGTCCTTTTATTACCGAAGTAGGTTTATTTTTCTCTTTTCTCACCACTATTTGCTTCA
                ****************  ******** ************ ********************

Nok1 K5 TAD3-1  AGGGTTTTTGATTCATTTAGCTTCCAAGTCAGAGATTTCTTTCTGCTCATTCATTTATAT
Nok1 K1 TAD3-2  AGGCTTTTTGATTCATTTAGCTTCCAAGGCAGAGATTTCTTTCTGCTCATTCATTTATAT
Nok1 K1 TAD3-3  AGGCTTTTTGATTCATTTAGCTTCCAAGGCAGAGATTTCTTTCTGCTCATTCATTTATAT
                *** ************************ *******************************

Nok1 K5 TAD3-1  TTTCAGTGCAATCTCATATTTCTGCTATAACTATGATAGGTATGCAAATATGCTGCGGTA
Nok1 K1 TAD3-2  TTTCAGTGCAATCTCATATTTCTGCTATAACTATGATAGGTATGCAAATATGCTGCGGTA
Nok1 K1 TAD3-3  TTTCAGTGCAATCTCATATTTCTGCTATAACTATGATAGGTATGCAAATATGCTGCGGTA
                ************************************************************

Nok1 K5 TAD3-1  TCCAAAGAAGAGTGGGAAGAACAAAGTAAGATATGGCCTACTTCATTTCATCCACCAACC
Nok1 K1 TAD3-2  TCCAAAGAAGAGTGGGAAGAACAAAGTAAGATATGGCCTACTTCATTTCATCCACCAACC
Nok1 K1 TAD3-3  TCCAAAGAAGAGTGGGAAGAACAAAGTAAGATATGGCCTACTTCATTTCATCCACCAACC
                ************************************************************

Nok1 K5 TAD3-1  TAGTAAGCTCCATGTCTAGAATCAGTTTTGTGACCAAAAGAGTCTTTATTATCTTTCTGG
Nok1 K1 TAD3-2  TAGTAAGCTCCATGTCTTGAATCAGTTTTGTGATCAAAGGAGTCTTTATTATC-------
Nok1 K1 TAD3-3  TAGTAAGCTCCATGTCTTGAATCAGTTTTGTGACCAAAGGAGTCTTTATTATC-------
                ***************** *************** **** **************

Nok1 K5 TAD3-1  TCTTTTTATTATATATGCTTTAGTCGTTCTAGAATAAT--CTTATTTCTTGATGGTTTCTA
Nok1 K1 TAD3-2  ----------ATCTAAGCTTTAGTCGTTTTGGAATAATATCTTATTTCTTGATGGTTTCTA
Nok1 K1 TAD3-3  ----------ATCTAAGCTTTAGTCGTTCTAGAATAATATCTTATTTCTTGATGGTTTCTA
                          ** ** ************ * *******  *********************

Nok1 K5 TAD3-1  TTGGTGCTCTTATCTGCATTGTAATAGCAATATATGTCTTTGTTTATAGTGCAACAGTCAA
Nok1 K1 TAD3-2  TTACTGCTCTTATCTGCATTGTAATAGCAATATATGTCTTTGTTTATAGTGCAACAGTCAA
Nok1 K1 TAD3-3  TTACTACTCTTATCTGCATTGTAATAGCAATATATGTCTTTGTTTATAGTGCAACAGTCAA
                **  * *******************************************************

Nok1 K5 TAD3-1  TGATTTTTTACTTTTTGCATTTGGCAATGATAGCAATATAGATGGCATCGGTGGGTTCAG
Nok1 K1 TAD3-2  TGATTTTTTACTTTTTTCATTTGGTAATGATAGCAATATAGATGGCATCGGTGGGTTCAG
Nok1 K1 TAD3-3  TGATTTTTTACTTTTTTCATTTGGTAATGATAGCAATATAGATGGCATCGGTGGGTTCAG
                **************** ******* ***********************************

Nok1 K5 TAD3-1  CGAGGAGGAAACACAATCAATCTGCAAGTTCATGAGAGTTGTTATTGATATGGCAGTATC
Nok1 K1 TAD3-2  CGAGGAGGAAACACAATCAATCTGCAAGTTCATGAGAGTTGTTATTGATATGGCAGTATC
Nok1 K1 TAD3-3  CGAGGAGGAAACACAATCAATGTGCAAGTTCATGAGAGTTGTTATTGATATGGCAGTATT
                ********************* ************************************** 

Nok1 K5 TAD3-1  TGGTCATACACCAGTAAGTATTTTCCATTGATCTACATAACGTCGATATCAAGTTTTAGA
Nok1 K1 TAD3-2  TGGTCATAAACCAGTAAGTATTTTCCGTTGATCTACATAACGTCGATATGAAGTTTTAGA
Nok1 K1 TAD3-3  TGGTCATAAACCAGTAAGTATTTTCCGTTGATCTACATAACGTCGATATGAAGTTTTAGA
                ******** ***************** ********************** **********
```

```
Nok1 K5 TAD3-1 CAACAGACCATTCATTTTTCATGTACCATTTGCTTTTTTTAGCGCCTCAATCCTTGTTCT
Nok1 K1 TAD3-2 CAACAGACCATTCATTTTTCACGTACCATTTGCTTGTTTTAGCGCCTCAATCCTTGTTTT
Nok1 K1 TAD3-3 CAACAGACCATTCATTTTTCACGTACCATTTGCTTGTTTTAGCGCCTCAATCCTTGTTCT
               ********************* ************* ********************** *

Nok1 K5 TAD3-1 CTTGGTAGAAAGATTTACCATCGTTTCATTACATAGAAAATGATCTCAATTTACCACATT
Nok1 K1 TAD3-2 CTTGGTCGAAAGATTTACCATCGTTTCATTACATAGAAAATGATCTCAATTTACCACATT
Nok1 K1 TAD3-3 CTTGGTCGAAAGATTTACCATCGTTTCATTACATAGAAAATGATCTCAATTTACCACATT
               ****** *****************************************************

Nok1 K5 TAD3-1 TCTAATGTACTTTTCAGCTTGTGAATGCTGCAGTGATAGTTGATCCTTCAGTTAGGCGAA
Nok1 K1 TAD3-2 TTTAATGTACTTTTCAGCTTGTGAATGCTGCAGTGATAGTTGATCCTTCAGTTAGGCGAA
Nok1 K1 TAD3-3 TTTAATGTACTTTTCAGCTTGTGAATGCTGCAGTGATAGTTGATCCTTCAGTTAGGCGAA
               * **********************************************************

Nok1 K5 TAD3-1 TAATAGCTAGTGAAACTGATCAAGTATATGCATCATCTGCTCCTCGTGACATGACTAGCG
Nok1 K1 TAD3-2 TAATAGCTAGTGAAACTGATCAAGTATATGCATCATCTGCTCCTCGTGACATGACTAGCG
Nok1 K1 TAD3-3 TAATAGCTAGTGAAACTGATCAAGTATATGCATCATCTGCTCCTCGTGACATGACTAGCG
               ************************************************************

Nok1 K5 TAD3-1 CAGAGACCAGGCYCTTCGAGGAAACAGGGGAAATATGTTTAAATGACACACTTGAAAAAC
Nok1 K1 TAD3-2 CAGAGACCAGGCCCTTCGAGGAAACAAGGGAAATATGCTTAAATGACACACTTGAAAAAC
Nok1 K1 TAD3-3 TAGAGACCAGGCCCTTCGAGGAAACAGGGGAAATATGCTTAAATGACACACTTGAAAAAC
                *********** ************* ********** **********************

Nok1 K5 TAD3-1 AGAATGGTTCATTGTCTGCTCTTTCTTGTCTGAATCCCTGGCAATGGAGTTTGCAGCCGC
Nok1 K1 TAD3-2 AGAATGGTTCATTGTCTGCTGTTTCTTGTCTGAATCCCTGGCAATGGAGTTTGCAGCCGC
Nok1 K1 TAD3-3 AGAATGGTTCATTGTCTGCTGTTTCTTGTCTGAATCCCTGGCAATGGAGTTTGCAGCCGC
               ******************** ***************************************

Nok1 K5 TAD3-1 ATGACACTGAAAATTGTAGCCAGTGGCATCCTCTTAGGCATGCTTCCATGGTTGCCATTG
Nok1 K1 TAD3-2 ATGACACTGAAAATTGTAGCGAAGGGCATCCTCTTAGGCATGCTTCCATGGTTGCCATTG
Nok1 K1 TAD3-3 ATGACACTGAAAATTGTAGCGAAGGGCATCCTCTTAGGCATGCTTCCATGGTTGCCATTG
               ******************** *  ************************************

Nok1 K5 TAD3-1 AATCCTCTTCTGCCAGAGATAGAAATCTGTTTCCCAATCCATCCAAGATTTTTGATCAGG
Nok1 K1 TAD3-2 AATCCTCTTCTGCCAGAGATAGAATTTTGTTTCCCAATCCATCCAAGATTTTTGATCAGG
Nok1 K1 TAD3-3 AATCCTCTTCTGCCAGAGATAGAATTTTGTTTCCCAATCCATCCAAGATTTTTGATCAGG
               ************************ * ********************************

Nok1 K5 TAD3-1 ATCATGTTCCGCCCTCAAATACAGATTCTCCGGCTAAAAAGCAGAAAACAAGCAGTCAGA
Nok1 K1 TAD3-2 ATCATGTTTTGCCCTCAAATACAGATTCTCCTGCTAAAAAGCAGAAAACAAGCAGTCAAA
Nok1 K1 TAD3-3 ATCATGTTTTGCCCTCAAATACAGATTCTCCGGCTAAAAAGCAGAAAACAAGTAGTCAAA
               ********  ********************* ******************** ***** *

Nok1 K5 TAD3-1 GTCCAGACGTAAGTTCATTCCAACCAAGTTTTTTAATTTCCTCCACATGCTTTGACTAGT
Nok1 K1 TAD3-2 GTCCAGAGGTAAGTTCATTCCAACCAAGTTTTTTAATTTCCTCCACATGCTTTGACTAGT
Nok1 K1 TAD3-3 GTCCAGAGGTAAGTTCATTCCAACCAAGTTTTTTAATTTCCTCCACATGCTTTGACTAGT
               ******* ****************************************************

Nok1 K5 TAD3-1 ACAGCATTCTCTCTTACACTTCTGAGTTATCGATAGGTCCAAAATGACAGCAGAGAAGAG
Nok1 K1 TAD3-2 ACAGCATTCTCTCTTACACTTCTGAGTTACCGATAGATCCAAAGTGACAGCAGAGAAGAG
Nok1 K1 TAD3-3 ACAGCATTCTCTCTTACACTTCTGAGTTACCGATAGATCCAAAGTGACAGCAGAGAAGAG
               ***************************** ****** ****** ****************

Nok1 K5 TAD3-1 ACTSTTAGAGATCCTTCAATGGAAAGGCCGTACCTCTGCACTGGTTATGACATTTTCCTC
Nok1 K1 TAD3-2 ACTCTTAGAGATCTTTCAATGGAAAGGCCGTACCTCTGCACTGGTTATGACATTTTCCTC
Nok1 K1 TAD3-3 ACTCTTAGAGATCTTTCAATGGAAAGGCCGTACCTCTGCACTGGTTATGACATTTTCCTC
               *** ********* **********************************************

Nok1 K5 TAD3-1 CTGTTGGAGCCTTGTACAATGTGAGTTTCTACATACATAT-TACCCTTTTAAACTCTGCA
Nok1 K1 TAD3-2 CTGTGGGAGCCTTGTACAATGTGAGTTTCTACATACATTATTACCCTTCTAACATCCGCA
Nok1 K1 TAD3-3 CTGTGGGAGCCTTGTACAATGTGAGTTTCTACATACATTATTACCCTTCTAACATCCGCA
               **** *********************************   ******* ***  ** ***

Nok1 K5 TAD3-1 AGTGTGTGTATCTAATAAAGTCGTGATATGTTCCCAAGGTGTGCTATGGCGCTTGTGCAT
Nok1 K1 TAD3-2 AGTTTGTGTATCTAATAAAGTCGTGATATCTTCTTAAGGTGTGCTATGGCGCTTGTGCAT
Nok1 K1 TAD3-3 AGTTTGTGTATCTAATAAAGTCGTGATATCTTCTTAAGGTGTGCTATGGCGCTTGTGCAT
               *** ************************* ***  *************************

Nok1 K5 TAD3-1 CAAAGAATAAAACGGATTTTCTATGCTTTTCCAAACACCACGGCAGGTGGTCTCGGGAGT
Nok1 K1 TAD3-2 CAAAGAATAAAACGGATTTTCTATGCTTTTCCAAACACCACGGCAGGTGGTCTCGGAAGT
Nok1 K1 TAD3-3 CAAAGAATAAAACGGATTTTCTATGCTTTTCCAAACACCACGGCAGGTGGTCTCGGGAGT
               ******************************************************** ***
```

```
Nok1 K5 TAD3-1 GTTCATAGACTTCAAGGGGAAAAGAGTTTGAACCATCATTATGCAGTGTTTAGAGTTTTG
Nok1 K1 TAD3-2 GTTCATAGACTTCAAGGGGAAAAGAGTTTGAACCATCATTATGCAGTGTTTAAAGTTTTG
Nok1 K1 TAD3-3 GTTCATAGACTTCAAGGGGAAAAGAGTTTGAACCATCATTATGCAGTGTTTAAAGTTTTG
               **************************************************** *******

Nok1 K5 TAD3-1 CTGCCTGATGACGCACTTAGACAAATGACCACGGTCTAATGCATTGTTGTAGTCGATTGA
Nok1 K1 TAD3-2 CTGCCTGATGACGCACTTAGACAAATGACCACGGTCTAATGCATTGTTGTAGTCGATTGA
Nok1 K1 TAD3-3 CTGCTTGATGACGCACTTAGACAAATGACCACGGTCCAATGCATTGTTGTAGTCGATTGA
               **** ******************************* ***********************

Nok1 K5 TAD3-1 TAATCAGGTTCTGGGTGAGAAAGCTAATTCTTTGTTCTTGTTCATTCCAAGAATCTGGAA
Nok1 K1 TAD3-2 TAAGCAGGTTCTGGG--TGAGAGCTAATTCTTTGTTCTTGTTCATTCCAAGAATCTTGTA
Nok1 K1 TAD3-3 TAAGCAGGTTCTGGG--TGAGAGCTAATTCTTTGTTCTTGTTCATTCCAAGAATCTTGTA
               *** ***********   ** *********************************** * *

Nok1 K5 TAD3-1 TCTTATTGAATTATTATGTAGACCAAAAA
Nok1 K1 TAD3-2 TCTTATTGAATTATTACGTAGACCAAAAA
Nok1 K1 TAD3-3 TCTTATTGAATTATTACGTAGACCAAAAA
               **************** ************
```
